# Hepatitis C virus induces oxidation and degradation of apolipoprotein B to enhance lipid accumulation and promote viral production

**DOI:** 10.1371/journal.ppat.1009889

**Published:** 2021-09-07

**Authors:** Bei Wang, Yue Zhu, Congci Yu, Chongyang Zhang, Qing Tang, He Huang, Zhendong Zhao

**Affiliations:** 1 NHC Key Laboratory of Systems Biology of Pathogens, Institute of Pathogen Biology, Chinese Academy of Medical Sciences & Peking Union Medical College, Beijing, China; 2 Clinical Immunology Center, Chinese Academy of Medical Sciences & Peking Union Medical College, Beijing, China; University of California, San Diego, UNITED STATES

## Abstract

Hepatitis C virus (HCV) infection induces the degradation and decreases the secretion of apolipoprotein B (ApoB). Impaired production and secretion of ApoB-containing lipoprotein is associated with an increase in hepatic steatosis. Therefore, HCV infection-induced degradation of ApoB may contribute to hepatic steatosis and decreased lipoprotein secretion, but the mechanism of HCV infection-induced ApoB degradation has not been completely elucidated. In this study, we found that the ApoB level in HCV-infected cells was regulated by proteasome-associated degradation but not autophagic degradation. ApoB was degraded by the 20S proteasome in a ubiquitin-independent manner. HCV induced the oxidation of ApoB via oxidative stress, and oxidized ApoB was recognized by the PSMA5 and PSMA6 subunits of the 20S proteasome for degradation. Further study showed that ApoB was degraded at endoplasmic reticulum (ER)-associated lipid droplets (LDs) and that the retrotranslocation and degradation of ApoB required Derlin-1 but not gp78 or p97. Moreover, we found that knockdown of ApoB before infection increased the cellular lipid content and enhanced HCV assembly. Overexpression of ApoB-50 inhibited lipid accumulation and repressed viral assembly in HCV-infected cells. Our study reveals a novel mechanism of ApoB degradation and lipid accumulation during HCV infection and might suggest new therapeutic strategies for hepatic steatosis.

## Author summary

Hepatitis C virus (HCV) infection induces the degradation of apolipoprotein B (ApoB), which is the primary apolipoprotein in low-density lipoprotein (LDL) and very low-density lipoprotein (VLDL). Impaired production and secretion of ApoB-containing lipoprotein is associated with an increase in hepatic steatosis. Thus, ApoB degradation might contribute to HCV infection-induced fatty liver. Here, we found that ApoB was not degraded through endoplasmic reticulum-associated degradation (ERAD) or autophagy, as reported previously. Instead, HCV infection induced ApoB oxidation through oxidative stress, and oxidatively damaged ApoB could be recognized and directly degraded by the 20S proteasome. We also found that ApoB was retrotranslocated from the endoplasmic reticulum (ER) to lipid droplets (LDs) for degradation. Through overexpression of ApoB-50, which can mediate the assembly and secretion of LDL and VLDL, we confirmed that ApoB degradation contributed to hepatocellular lipid accumulation induced by HCV infection. Additionally, expression of ApoB-50 impaired HCV production due to the observed decrease in lipid accumulation. In this study, we identified new mechanisms of ApoB degradation and HCV-induced lipid accumulation, and our findings might facilitate the development of novel therapeutic strategies for HCV infection-induced fatty liver.

## Introduction

Hepatitis C virus (HCV) has infected approximately 71 million people, and 1.7 million new infections occur annually [1]. If untreated, 10% to 20% of patients develop liver cirrhosis, which progresses to hepatocyte carcinoma in 1% to 4% of these patients [2]. HCV is an enveloped, single-stranded, positive-sense RNA virus that is a member of the Flaviviridae family. The HCV genome consists of 9.6 kb of RNA encoding a single polyprotein that is processed by viral proteases and cellular signal peptidases to produce three structural proteins (core, envelope 1 [E1], and E2) and seven nonstructural proteins (p7, nonstructural protein 2 [NS2], NS3, NS4A, NS4B, NS5A, and NS5B) [3]. Similar to that of other viruses, the entire life cycle of HCV relies on various cellular organelles and host factors [4].

Chronic HCV infection is associated with an increase in hepatic steatosis and a decrease in serum levels of total cholesterol, low-density lipoprotein (LDL), and very low-density lipoprotein (VLDL) cholesterol [5]. These changes suggest that lipids may play an important role in the life cycle of HCV. The HCV replication organelle (the membranous web) is a dynamic complex associated with the endoplasmic reticulum (ER) and lipid droplets (LDs). The assembly of HCV particles occurs on the ER-LD interface; the HCV assembly pathway appears to share numerous features with the LDL/VLDL assembly pathway, and HCV virions are secreted as hybrid lipoviral particles (LVPs) [6,7]. The lipid content of LVPs is not only important for virion assembly but also involved in the entry step [8,9].

Apolipoprotein B (ApoB) is the primary apolipoprotein in chylomicrons, LDL particles, and VLDL particles. There are two forms of ApoB, ApoB-100 (produced in the liver) and ApoB-48 (produced in the small intestine), both of which are encoded by the same gene. Hepatic ApoB-100 contains 4536 amino acids and is essential for the assembly of VLDL. ApoB-48 has a molecular mass 52% lower than that of ApoB-100 and is necessary for the formation of chylomicrons [10]. Dysfunction of the quality control mechanisms for ApoB may induce hepatic lipotoxicity, which is relevant to fatty liver disease, hepatic insulin resistance, and inflammation. Under physiological conditions, poorly lipidated nascent ApoB can be degraded by ER-associated degradation (ERAD) without inducing ER stress to maintain cellular homeostasis [11–13]. Retrotranslocation and degradation of nascent ApoB by ERAD require Gp78 and p97 [14–16]. In contrast, lipidated ApoB can be subject to selective autophagy [17,18]. Lipidated ApoB is translocated from the ER membrane to ER-associated LDs for degradation, a process that requires Derlin-1[17]. Both mechanisms are ubiquitin-dependent.

HCV infection is associated with a decrease in the serum level of ApoB [19,20]. Mancone et al. found that HCV infection induces proteasomal degradation of ApoB in hepatocytes [21], which may cause a decrease in ApoB secretion. However, the mechanism underlying HCV infection-induced ApoB degradation is incompletely elucidated. In this study, we investigated the detailed mechanism underlying the degradation of ApoB during HCV infection. We further investigated whether and how the degradation of ApoB impacts HCV replication.

## Results

### The protein level of ApoB was decreased in HCV-infected cells

**Fig 1:**
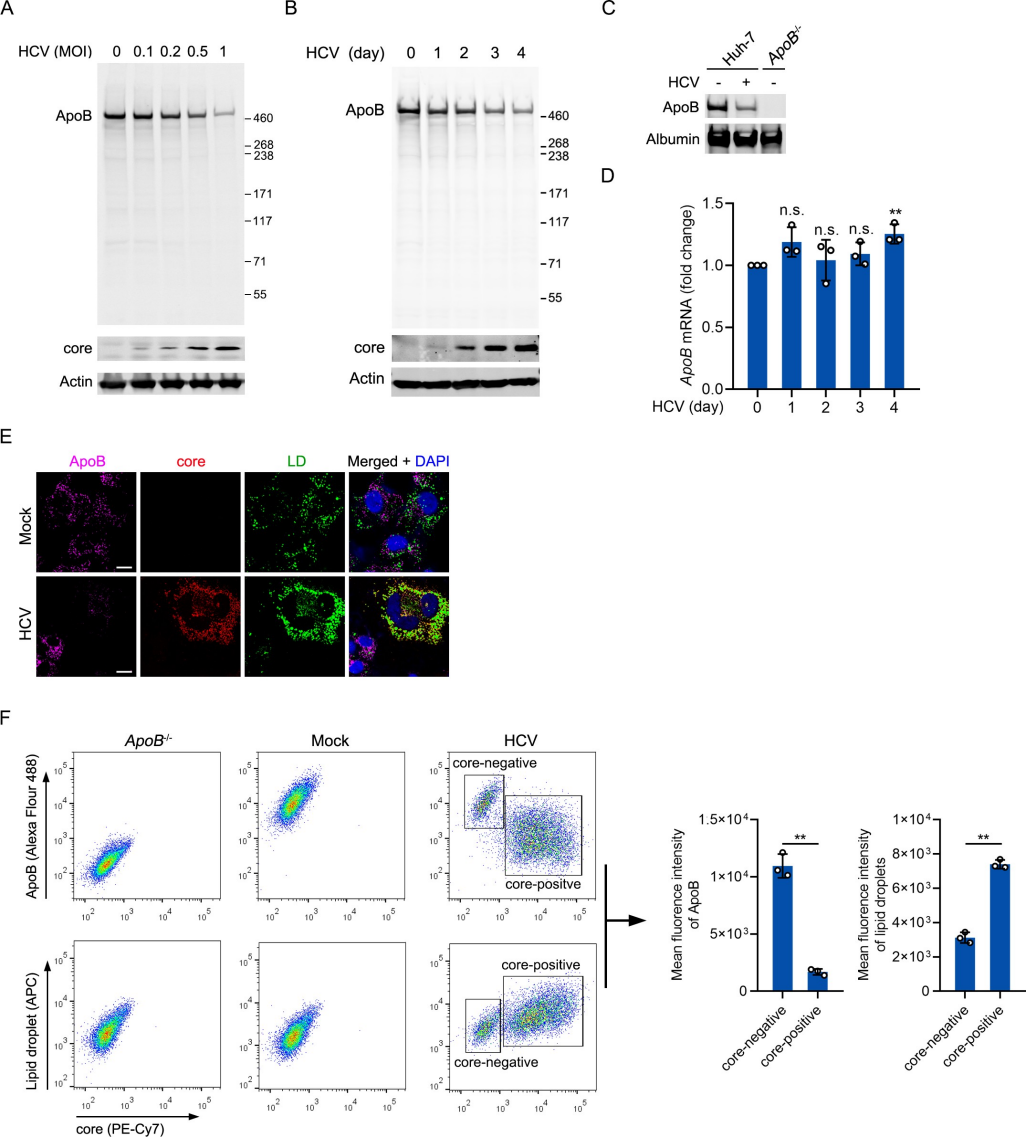
The protein level of ApoB was decreased in HCV-infected cells. (A) Huh-7 cells were infected with HCV at different MOIs. After 96 hours, the protein levels of ApoB and HCV core proteins were analyzed by western blotting. Actin was used as the loading control. (B) Huh-7 cells were infected with HCV (MOI = 1). The protein levels of ApoB and HCV core proteins were analyzed by western blotting at the indicated timepoints after infection. Actin was used as the loading control. (C) The protein level of ApoB in culture supernatants of mock-infected, HCV-infected (MOI = 1) Huh-7 cells or *ApoB*^-/-^ cells was analyzed by western blotting at 4 days post infection. Albumin was used as the loading control. (D) qPCR analysis of the mRNA level of *ApoB* in cells in B. The results are presented as fold changes in the mRNA level of *ApoB* relative to that of *GAPDH*. (E) Immunostaining was performed with anti-ApoB and anti-core antibodies in mock- or HCV-infected (MOI = 1) Huh-7 cells at 4 days post infection. LDs were stained with BODIPY 493/503. Nuclei were stained with DAPI. Fluorescence signals were visualized by laser confocal microscopy. LD, lipid droplet. (F) Flow cytometric analysis was performed in *ApoB*^-/-^ cells and mock- or HCV-infected (MOI = 0.5) cells at 4 days post infection. The staining of ApoB, core, and LDs is described in the Methods. The data are shown as the means ± SDs of *n* = 3 biological repeats. The statistical significance was determined by unpaired two-sided Student’s *t*-tests. n.s., not significant. ** *P* < 0.01.

The levels of serum ApoB were reported to be negatively correlated with the HCV viral load and steatosis [19,20]. We analyzed the protein level of ApoB in Huh-7 cells infected with HCV at different multiplicities of infection (MOIs) and found by western blot analysis that the ApoB level decreased in a dose-dependent manner (Fig 1A). We also analyzed the level of ApoB in HCV-infected cells over a 4-day time course and found that ApoB was significantly decreased at days 3 and 4 after HCV infection (Fig 1B). In addition, the level of secreted ApoB in the culture supernatant was reduced (Fig 1C). We investigated whether the reduction in ApoB protein was induced by the decrease in *ApoB* transcription and found that the mRNA level of *ApoB* was slightly increased at 4 days after HCV infection (Fig 1D). Confocal microscopy analysis also showed that the ApoB level decreased and LD accumulation increased in HCV-infected cells (Fig 1E). The results were validated by flow cytometric analysis (Fig 1F). We found that core-positive cells had less ApoB and more LDs than core-negative cells.

### The degradation of ApoB was mediated by the proteasome but not autophagy in HCV-infected cells

**Fig 2:**
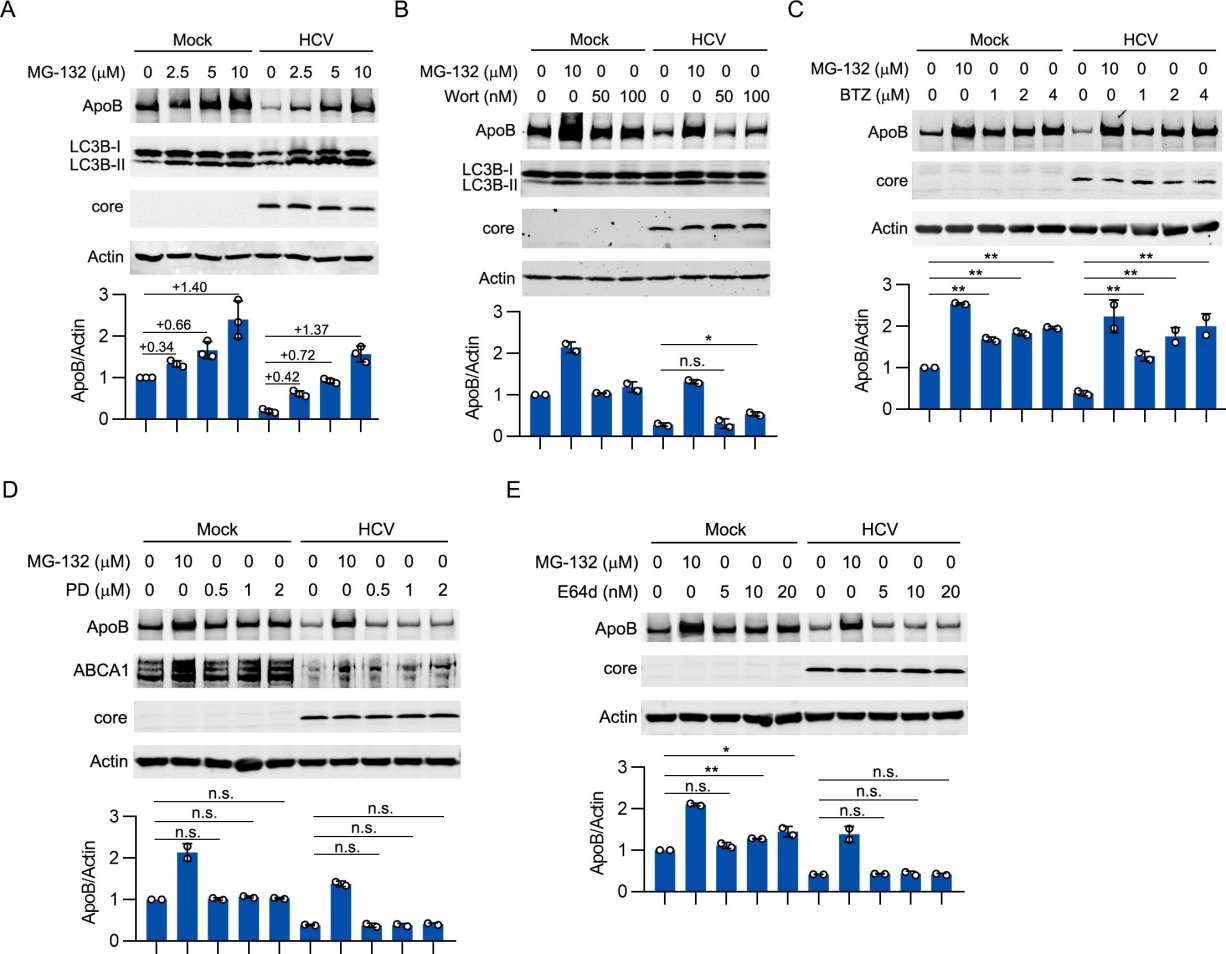
ApoB was degraded by the proteasome in HCV-infected cells. Huh-7 cells were infected with HCV (MOI = 1) for 4 days followed by treatment with MG-132 (A), wortmannin (B), bortezomib (C), PD151746 (D), or E64d (E) for 12 hours. The levels of the indicated proteins were analyzed by western blotting. Actin was used as the loading control. Wort, wortmannin. BTZ, bortezomib. PD, PD151746. The data are presented as the means ± SDs from densitometry analyses of *n* = 2 or 3 independent experiments, and representative gels from each specific assay are shown. The statistical significance was determined by unpaired two-sided Student’s *t*-tests. n.s., not significant. * *P* < 0.05. ** *P* < 0.01.

We then analyzed whether this reduction resulted from the degradation of ApoB. Both the proteasome and autophagy are reported to participate in ApoB degradation [15,17], and our group found that HCV infection induced complete autophagy [22,23]. Therefore, we employed proteasome and autophagy inhibitors to analyze whether these two pathways were involved in the degradation of ApoB. As shown in Fig 2A, MG-132, a widely used proteasome inhibitor, substantially inhibited the HCV-induced reduction in the ApoB level. MG-132 also inhibited autolysosomal degradation, as indicated by the increase in LC3-II, which was degraded in autolysosomes. Wortmannin (an autophagy inhibitor) inhibited HCV-induced autophagy, as indicated by the decrease in LC3-II formation (Fig 2B). However, treatment with a high concentration of wortmannin only slightly restored the ApoB level. These results suggested that the proteasome plays a dominant role in the degradation of ApoB in HCV-infected cells.

Because MG-132 also inhibits the activity of calpain and cysteine proteases as previously reported [24], we used a more specific proteasome inhibitor (bortezomib) [25] to determine whether ApoB is degraded by the proteasome during HCV infection. As shown in Fig 2C, bortezomib restored the ApoB level in mock- and HCV-infected cells in a dose-dependent manner. Furthermore, inhibitors of calpain and cysteine proteases were used to analyze whether these proteases degraded ApoB. A calpain inhibitor, PD151746 [26], did not inhibit the degradation of ApoB in mock- or HCV-infected cells (Fig 2D). ABCA1, which is proteolytically degraded by calpain, was used as a positive control [27]. The protein level of ABCA1 was increased by PD151746 in mock-infected cells. Interestingly, HCV infection decreased the ABCA1 level. In addition, a cysteine protease inhibitor, E-64d [28], slightly inhibited ApoB degradation in noninfected cells but did not restore the ApoB level in HCV-infected cells (Fig 2E). These data suggested that HCV infection induced proteasomal degradation of ApoB.

### ERAD did not participate in HCV infection-induced degradation of ApoB

**Fig 3:**
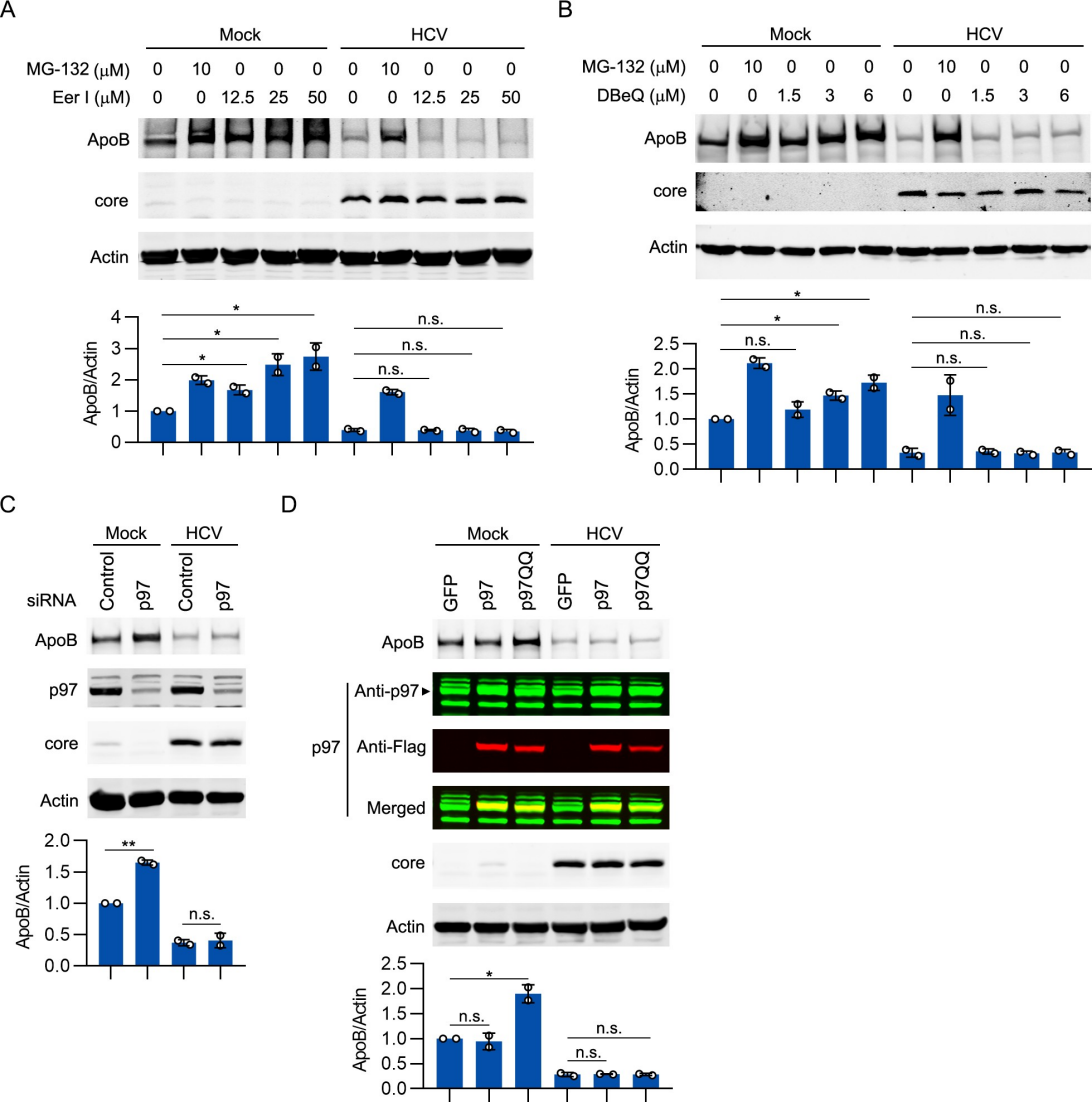
ERAD did not participate in HCV-induced ApoB degradation. (A, B) Huh-7 cells were infected with HCV (MOI = 1) for 4 days prior to treatment with MG-132, Eer I, or DBeQ for 12 hours. The levels of ApoB, Herp, and core were analyzed by western blotting. Actin was used as the loading control. (C) Huh-7 cells were infected with HCV (MOI = 1) for 48 hours prior to transfection with p97 siRNA for 2 days. The levels of ApoB, p97, and core were analyzed by western blotting. Actin was used as the loading control. (D) Huh-7 cells were infected with HCV (MOI = 1) for 2 days prior to transduction with GFP, a Flag-tagged wild type of p97, or a Flag-tagged dominant-negative p97 mutant (p97QQ) for 2 days. The levels of ApoB, p97, and core were analyzed by western blotting. Actin was used as the loading control. The data are presented the means ± SDs from densitometry analyses of *n* = 2 independent experiments, and representative gels from each specific assay are shown. The statistical significance was determined by unpaired two-sided Student’s *t*-tests. n.s., not significant. * *P* < 0.05. ** *P* < 0.01.

As mentioned above, the turnover of newly synthesized ApoB is mediated by ERAD, a proteasome-mediated degradation pathway. We then analyzed whether the ERAD pathway was enhanced in HCV-infected cells to degrade ApoB. Eeyarestatin I (Eer I) is an ERAD inhibitor that can inhibit the activity of p97 and translocation mediated by Sec61 [29]. As shown in Fig 3A, Eer I increased the protein level of ApoB in mock-infected cells. However, Eer I did not restore the ApoB level in HCV-infected cells. Moreover, DBeQ, a specific inhibitor of p97 [30], did not restore the ApoB level in HCV-infected cells (Fig 3B). Furthermore, transfection with p97 siRNA inhibited the degradation of ApoB in noninfected cells but not in HCV-infected cells (Fig 3C). Overexpression of a dominant-negative mutant of p97 (p97QQ) [31] but not overexpression of wild-type p97 inhibited ApoB degradation in mock-infected cells (Fig 3D). However, overexpression of p97QQ did not increase the protein level of ApoB in HCV-infected cells. These data suggested that ERAD is not involved in HCV infection-induced degradation of ApoB.

### ApoB was degraded by the 20S proteasome in HCV-infected cells in a ubiquitin-independent manner

**Fig 4:**
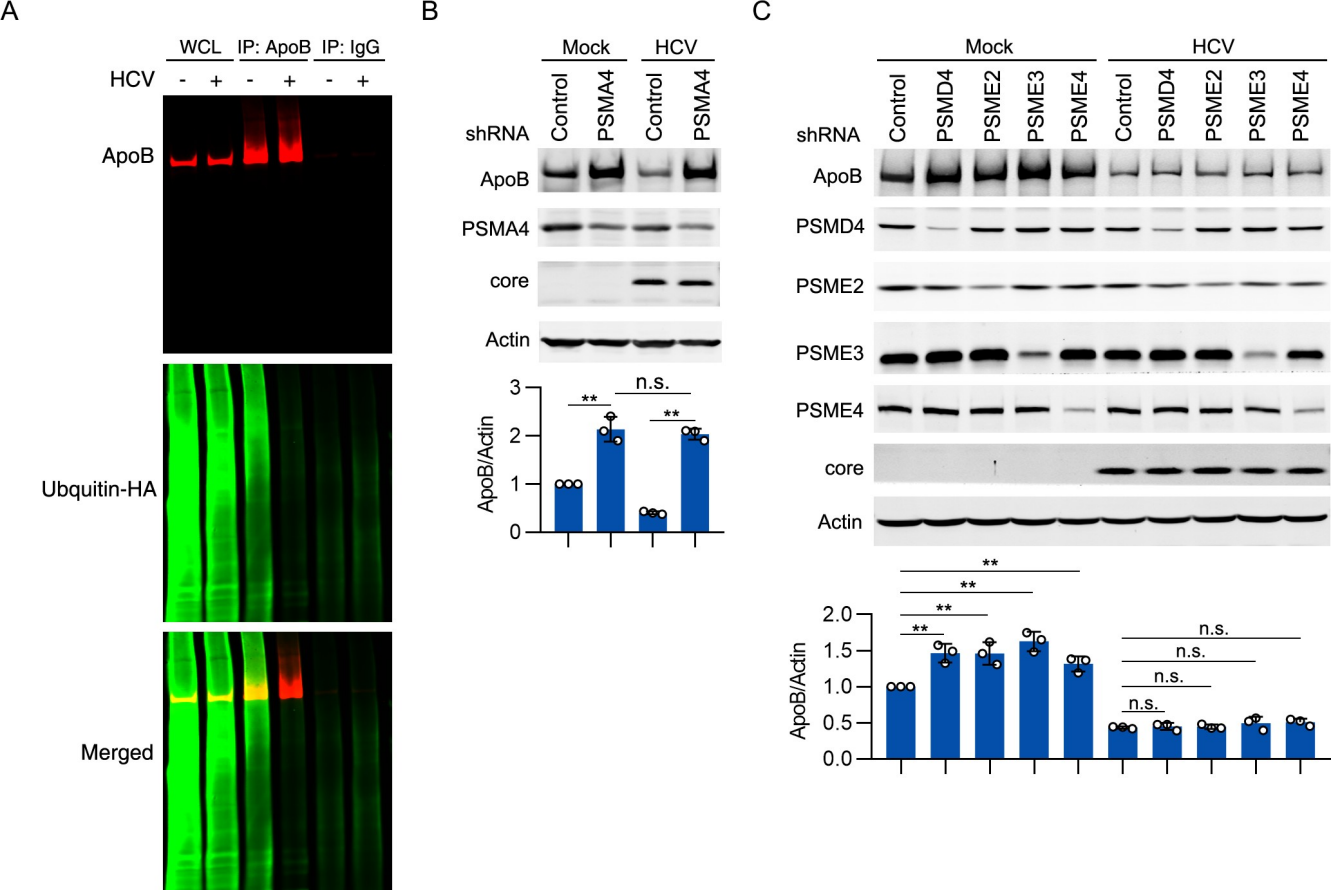
ApoB was degraded by the proteasome in a ubiquitin-independent manner in Huh-7 cells. (A) Huh-7 cells were infected with HCV (MOI = 1) for 2 days prior to transduction with HA-tagged ubiquitin (MOI = 1) for 2 days. At 4 days post HCV infection, the cells were treated with 10 μM MG-132 for 12 hours. ApoB was immunoprecipitated under denaturing conditions, and the level of ubiquitin was analyzed by western blotting. WCL, whole-cell lysate. (B) Huh-7 cells were infected with HCV (MOI = 1) for 2 days prior to transduction with PSMA4 shRNA for 2 days. The protein levels of ApoB, PSMA4, and core proteins were analyzed by western blotting. Actin was used as the loading control. (C) Huh-7 cells were infected with HCV (MOI = 1) for 2 days prior to transduction with PSMD4, PSME2, PSME3, or PSME4 shRNA for 2 days. The protein levels of ApoB, PSMD4, PSME2, PSME3, PSME4, and core were analyzed by western blotting. Actin was used as the loading control. The data in B and C are presented as the means ± SDs from densitometry analyses of *n* = 3 independent experiments, and representative gels from each specific assay are shown. The statistical significance was determined by unpaired two-sided Student’s *t*-tests. n.s., not significant. ** *P* < 0.01.

Proteasomal degradation can occur in a ubiquitin-dependent or ubiquitin-independent manner. We proceeded to investigate whether ApoB is ubiquitinated before proteasomal degradation. HA-tagged ubiquitin was overexpressed in mock-infected and HCV-infected cells, and the cells were then treated with MG-132. ApoB was immunoprecipitated, and ubiquitination of ApoB was analyzed. As shown in Fig 4A, ubiquitination of ApoB was substantially decreased in HCV-infected cells compared with mock-infected cells, suggesting that ApoB is degraded in a ubiquitin-independent manner.

The core of the proteasome is a four-ringed, barrel-shaped protein complex called the 20S proteasome. The 20S proteasome can function independently or can bind to several regulator complexes that modify its substrate selection and proteolytic activity. These regulators include the 19S, PA28αβ (11S proteasome), PA28γ, and PA200 regulators [32]. We investigated which type of proteasome-related component participates in ApoB degradation. Knockdown of the PSMA4 subunit of the 20S proteasome greatly increased the protein level of ApoB in both mock- and HCV-infected cells (Fig 4B), confirming that the proteasome participates in the degradation of ApoB. Silencing the 19S proteasome subunits PSMD4, PSME2 (PA28β), PSME3 (PA28γ), or PA200 inhibited the degradation of ApoB in mock-infected cells. However, knockdown of these genes did not restore the ApoB level in HCV-infected cells (Fig 4C). These results suggested that the 20S proteasome degrades ApoB independently of these proteasome regulators.

### The oxidation of ApoB promoted its degradation by the 20S proteasome in HCV-infected cells

**Fig 5:**
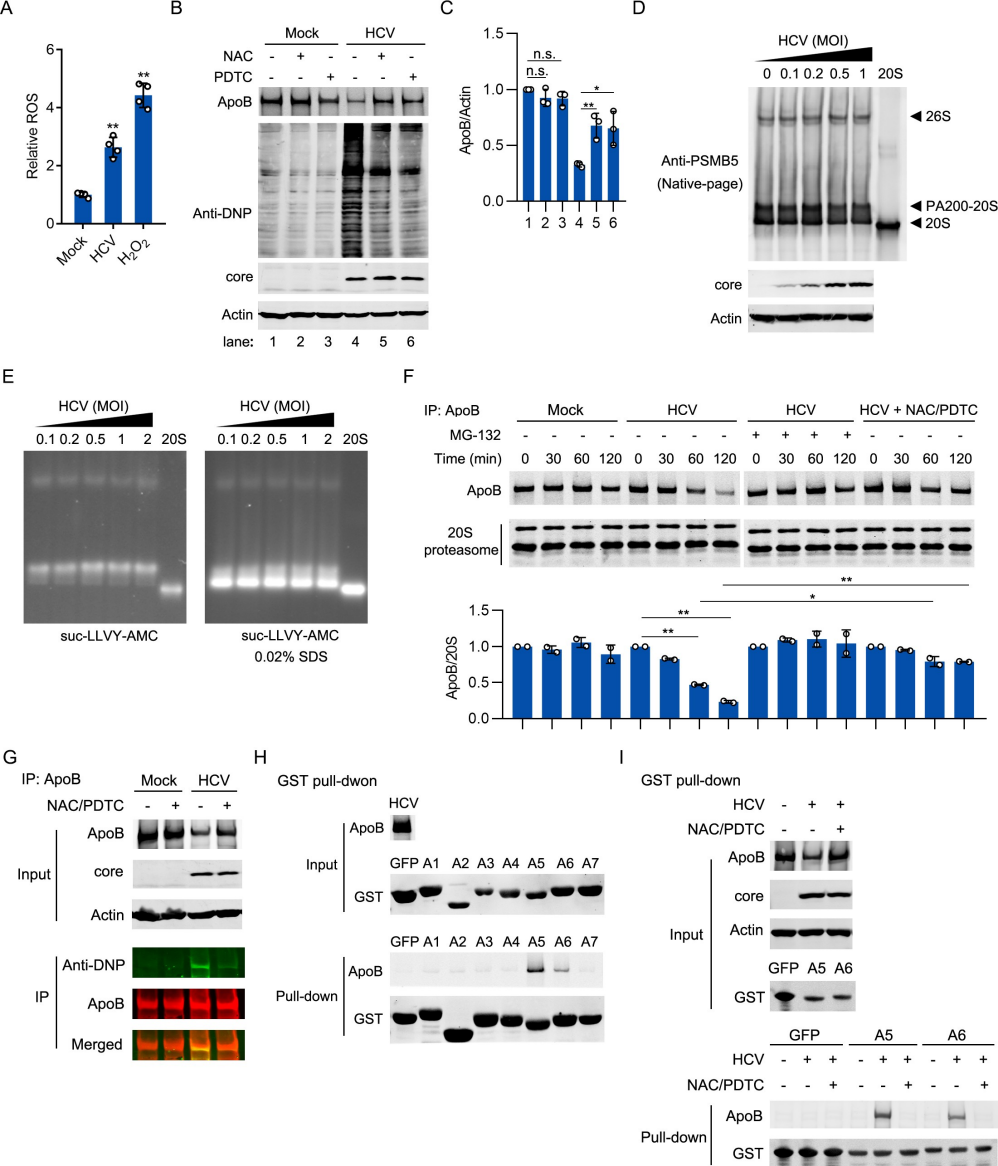
Oxidized ApoB was recognized by the 20S proteasome for degradation. (A) Huh-7 cells were infected with HCV (MOI = 1) for 4 days or treated with 1 μM H_2_O_2_ for 24 hours. Intracellular ROS levels were analyzed using a Fluorometric Intracellular ROS Kit. (B and C) Huh-7 cells were infected with HCV (MOI = 1) for 4 days prior to treatment with 1 mM NAC or 100 μM PDTC for 12 hours. ApoB was immunoprecipitated, and carbonyl groups generated by oxidation were derivatized to DNP and detected by western blotting with an anti-DNP antibody. The protein levels of ApoB and the core were also analyzed. Actin was used as the loading control. (D) Huh-7 cells were infected with HCV at different MOIs. Cell lysates were separated on a native-PAGE gel. Proteasomes were detected by western blotting with an anti-PSMB5 antibody. (E) Huh-7 cells were infected with HCV at different MOIs. Cell lysates were separated on a native-PAGE gel. The proteolytic activities of the 26S proteasome and the 20S proteasome (in the presence of 0.02% SDS) were analyzed with the proteasome substrate suc-LLAV-AMC. The stained gel was analyzed using a UV trans-illuminator at 365 nm wavelength. (F) ApoB was immunoprecipitated from mock-infected, HCV-infected (MOI = 1), and HCV-infected NAC/PDTC-treated cells prior to treatment with MG-132. Immunoprecipitated ApoB was incubated with purified 20S proteasome for the indicated timepoints in vitro. ApoB and the 20S proteasome in the reaction mixtures were analyzed by immunoblotting. (G) ApoB was immunoprecipitated from mock-infected and HCV-infected cells (MOI = 1) at day 4 after infection. Protein oxidation was analyzed as described in Methods. (H) A GST pulldown assay was performed to evaluate the interaction of the 20S proteasome subunit PSMA1-PSMA7 with ApoB from HCV-infected (MOI = 1) and MG-132-treated Huh-7 cells. (I) A GST pulldown assay was performed to evaluate the interaction of the 20S proteasome subunits PSMA5 and PSMA6 with ApoB from mock-infected, HCV-infected (MOI = 1), or HCV-infected Huh-7 cells treated with MG-132. The data in C and F are presented as the means ± SDs from densitometry analyses of *n* = 2 or 3 independent experiments, and representative gels from each specific assay are shown. The statistical significance was determined by unpaired two-sided Student’s *t*-tests. n.s., not significant. * *P* < 0.05. ** *P* < 0.01.

HCV infection has been reported to induce oxidative stress [33]. Oxidative stress can increase the amount of the 20S proteasome and promote its activity [34]. Thus, we hypothesized that the degradation of ApoB might be associated with oxidative stress induced by HCV infection. HCV infection indeed increased reactive oxygen species (ROS) levels and induced protein oxidation (Fig 5A and 5B). In addition, the oxidative stress inhibitors N-acetylcysteine (NAC) and pyrrolidine dithiocarbamate (PDTC) [35] inhibited protein oxidation and ApoB degradation in HCV-infected cells (Fig 5B and 5C). However, neither the amount nor the proteolytic activity of the 20S proteasome was changed in HCV-infected cells (Fig 5D and 5E).

The results of an in vitro degradation assay showed that the purified 20S proteasome degraded ApoB isolated from HCV-infected cells but not ApoB isolated from mock-infected cells or NAC/PDTC-treated HCV-infected cells (Fig 5F). These results suggested that the 20S proteasome-mediated degradation of ApoB was related to oxidative stress. Because oxidized proteins can be targeted for degradation by the 20S proteasome[36–38], we speculated that ApoB might be oxidized and targeted for degradation by the 20S proteasome in HCV-infected cells. Indeed, ApoB was oxidized in HCV-infected cells, and treatment of these cells with oxidative stress inhibitors (NAC and PDTC) inhibited its oxidation (Fig 5G). However, NAC and PDTC did not impact the protein level of ApoB in mock-infected cells. These results suggested that the degradation of ApoB might be associated with oxidative stress induced by HCV infection.

### Oxidized ApoB interacted with the PSMA5 and PSMA6 subunits of the 20S proteasome

We then proceeded to investigate the mechanism by which oxidized ApoB is degraded by the 20S proteasome. It has been reported that protein substrates can be directly recognized by the 20S proteasome for degradation [37–39]. Therefore, we analyzed whether ApoB can interact with the α subunits of the 20S proteasome. The results of pulldown assays showed that ApoB interacted with PSMA5 and PSMA6 but not the other five α subunits in HCV-infected cells (Fig 5H). In contrast, no interaction was detected between PSMA5 or PSMA6 and ApoB immunoprecipitated from mock-infected cells (Fig 5I). Moreover, the interaction between PSMA5 or PSMA6 and ApoB immunoprecipitated from HCV-infected cells was abolished by treatment with NAC/PDTC. These data suggested that oxidation of ApoB might promote its recognition and degradation by the 20S proteasome.

### ApoB was retrotranslocated from the ER lumen to LDs for degradation

**Fig 6:**
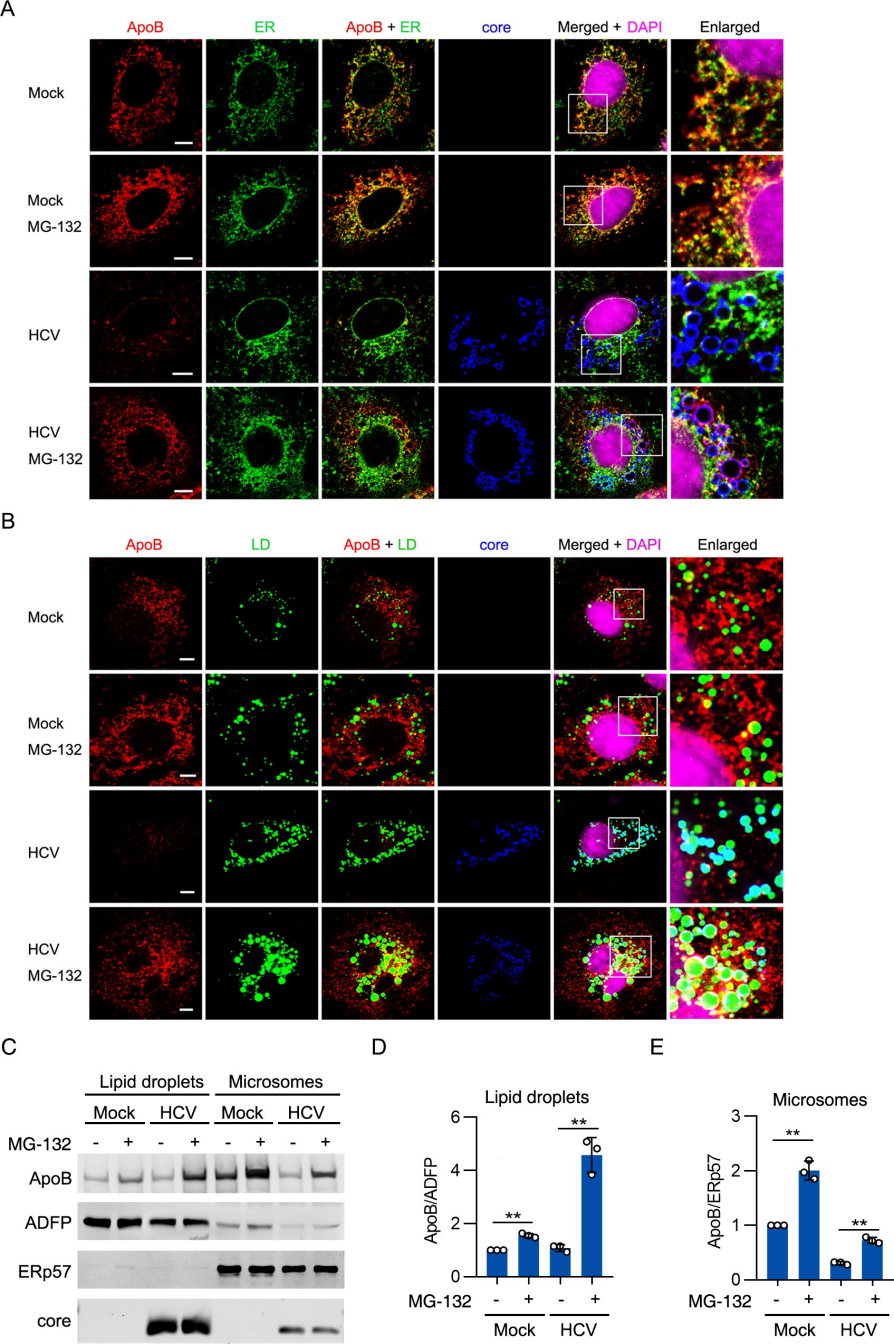
ApoB was degraded on ER-associated LDs in HCV-infected cells. (A, B) Huh-7 cells were infected with HCV (MOI = 1) for 4 days followed by treatment with MG-132 for 12 hours. Immunostaining was performed with antibodies against ApoB, core, and calnexin (an ER marker). LDs were stained with BODIPY 493/503. Nuclei were stained with DAPI. Fluorescence signals were visualized by laser confocal microscopy. Scale bars, 5 μM. (C-E) Huh-7 cells were infected with HCV (MOI = 1) for 4 days followed by treatment with MG-132 for 12 hours. The protein level of ApoB was analyzed in LD fractions and microsomal fractions. A LD marker, ADFP, and an ER marker, ERp57, were used as loading controls. The data in D and E are presented as the means ± SDs of densitometry values from *n* = 3 independent experiments, and representative gels from each specific assay are shown in C. The statistical significance was determined by unpaired two-sided Student’s *t*-tests. ** *P* < 0.01.

The subcellular location at which proteasomal degradation of ApoB occurs in HCV-infected cells remains unknown. ApoB was mainly distributed in the ER in untreated Huh-7 cells (Fig 6A). Because ERAD of ApoB occurs under physiological conditions, ApoB was increased and colocalized with ER after treatment with MG-132. In HCV-infected cells, in addition to exhibiting colocalization with the ER, a portion of ApoB assembled into a circular shape and was associated with the core protein after treatment with MG-132. Because the HCV core protein is well established to be distributed on cytosolic LDs [40–42], it can be used as a surface marker of cytosolic LDs (Fig 1E). We hypothesized that ApoB might be distributed on cytosolic LDs in HCV-infected cells after treatment with MG-132. After MG-132 treatment, a large portion of ApoB was observed on LDs in HCV-infected cells, but only a small amount of ApoB was distributed on LDs in mock-infected cells (Fig 6B). Because we could not simultaneously stain ApoB, the ER, LDs, and the core protein, we could not analyze the colocalization of ApoB, the ER, and LDs in HCV-infected cells. However, because the core protein is distributed on LDs, staining of ApoB, the ER, and the core protein indicated the distribution of ApoB on ER-associated LDs in HCV-infected cells after treatment with MG-132 (Fig 6A).

To validate that ApoB was degraded on cytosolic LDs in HCV-infected cells, we isolated LDs and microsomes and analyzed the protein level of ApoB. After treatment with MG-132, the level of ApoB was markedly increased in the LD fraction and the microsomal fraction in HCV-infected cells. However, the ApoB level was slightly increased in the LD fraction and significantly increased in the microsomal fraction in mock-infected cells after MG-132 treatment (Fig 6C–6E). These results suggested that ApoB might be retrotranslocated from the ER lumen to LDs for degradation.

### Derlin-1 but not gp78 participated in ApoB degradation

**Fig 7:**
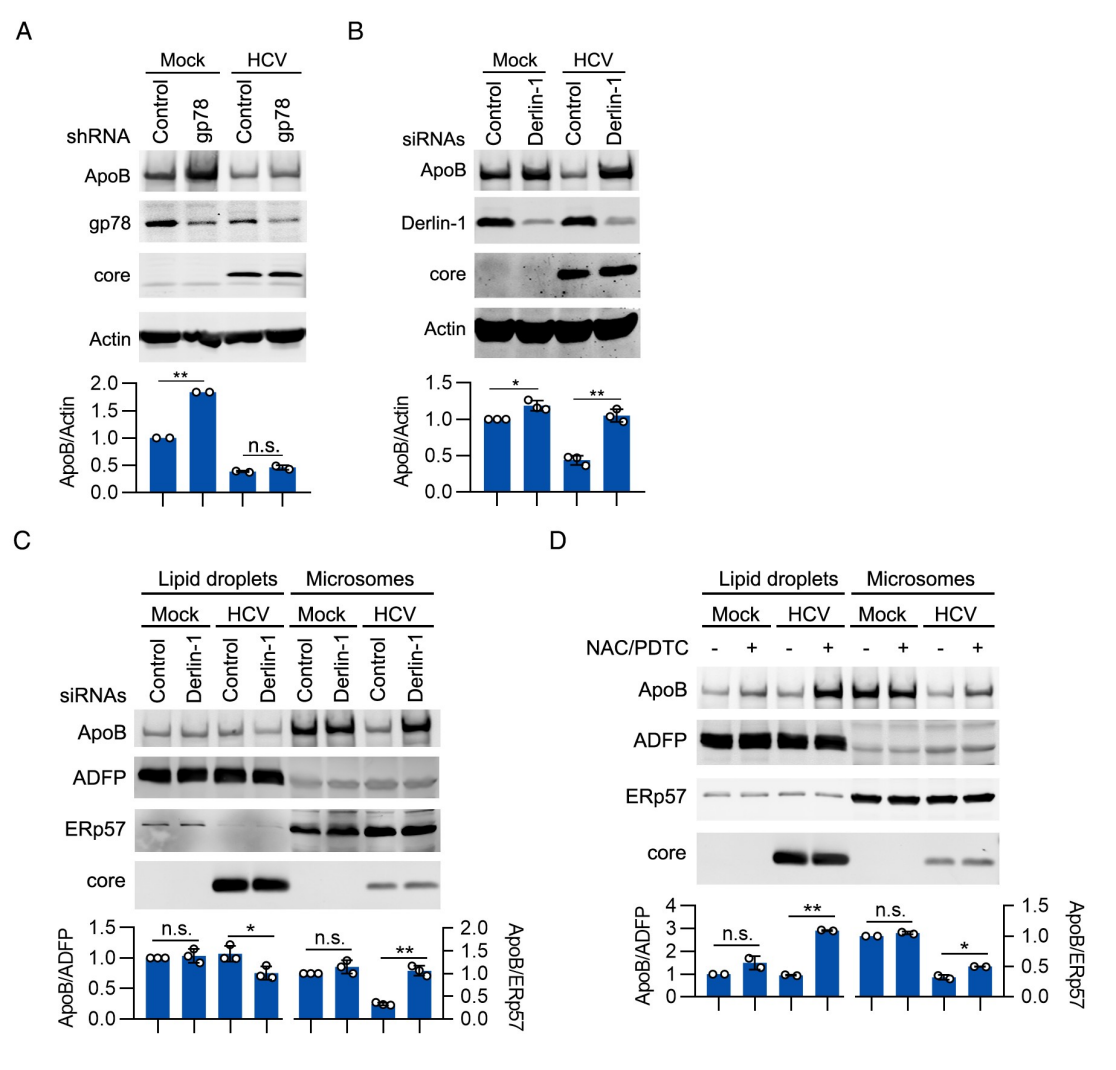
Derlin-1 but not gp78 participated in ApoB degradation. (A) Huh-7 cells were infected with HCV (MOI = 1) for 2 days followed by transduction with gp78 shRNA for 2 days. The protein levels of ApoB, gp78, and core were analyzed by western blotting at 4 days after infection. Actin was used as the loading control. (B) Huh-7 cells were infected with HCV (MOI = 1) for 2 days followed by transfection with Derlin-1 siRNAs for 2 days. The protein levels of ApoB, Derlin-1, and core proteins were analyzed by western blotting at 4 days after infection. Actin was used as the loading control. (C) Huh-7 cells were infected with HCV (MOI = 1) for 2 days followed by transfection with Derlin-1 siRNAs for 2 days. The protein level of ApoB was analyzed in LD fractions and microsomal fractions. ADFP and ERp57 were used as loading controls. (D) Huh-7 cells were infected with HCV for 4 days prior to treatment with 1 mM NAC and 100 μM PDTC for 12 hours. The protein level of ApoB was analyzed in LD fractions and microsomal fractions. ADFP and ERp57 were used as loading controls. The data are presented as the means ± SDs from densitometry analyses of *n* = 2 or 3 independent experiments, and representative gels for each specific assay are shown. The statistical significance was determined by unpaired two-sided Student’s *t*-tests. n.s., not significant. * *P* < 0.05. ** *P* < 0.01.

During ERAD, newly synthesized ApoB should be retrotranslocated from the ER lumen to the cytoplasm before degradation. This pathway requires gp78 for retrotranslocation and ubiquitination [43]. However, Suzuki et al. identified an alternative pathway in which retrotranslocation and degradation of lipidated “ApoB crescents” at LDs required Derlin-1 and UBXD8 [17]. We sought to determine whether ApoB is retrotranslocated through these pathways. As shown in Fig 7A, knockdown of gp78 increased the ApoB level in mock-infected cells but not in HCV-infected cells. HCV infection decreased the protein level of gp78, suggesting that HCV infection might impair gp78-associated ERAD. Knockdown of Derlin-1 moderately inhibited ApoB degradation in mock-infected cells but substantially increased the ApoB level in HCV-infected cells (Fig 7B). We found that the level of ApoB was significantly increased in the microsomal fraction but decreased in the LD fraction in HCV-infected cells after Derlin-1 siRNA transfection (Fig 7C). These results suggested that Derlin-1 but not gp78 participated in the retrotranslocation and degradation of ApoB during HCV infection.

Another question raised was whether ApoB oxidation induced retrotranslocation. We found that the level of ApoB was significantly increased in the LD fraction but slightly increased in the microsomal fraction in HCV-infected cells after treatment with NAC/PDTC (Fig 7D), suggesting that retrotranslocation of ApoB might not be induced by oxidation. We did not investigate how HCV infection induced the retrotranslocation of ApoB in this study. The mechanism might be revealed in the future.

### Knockdown of ApoB increased the contents of LDs and promoted HCV assembly

**Fig 8:**
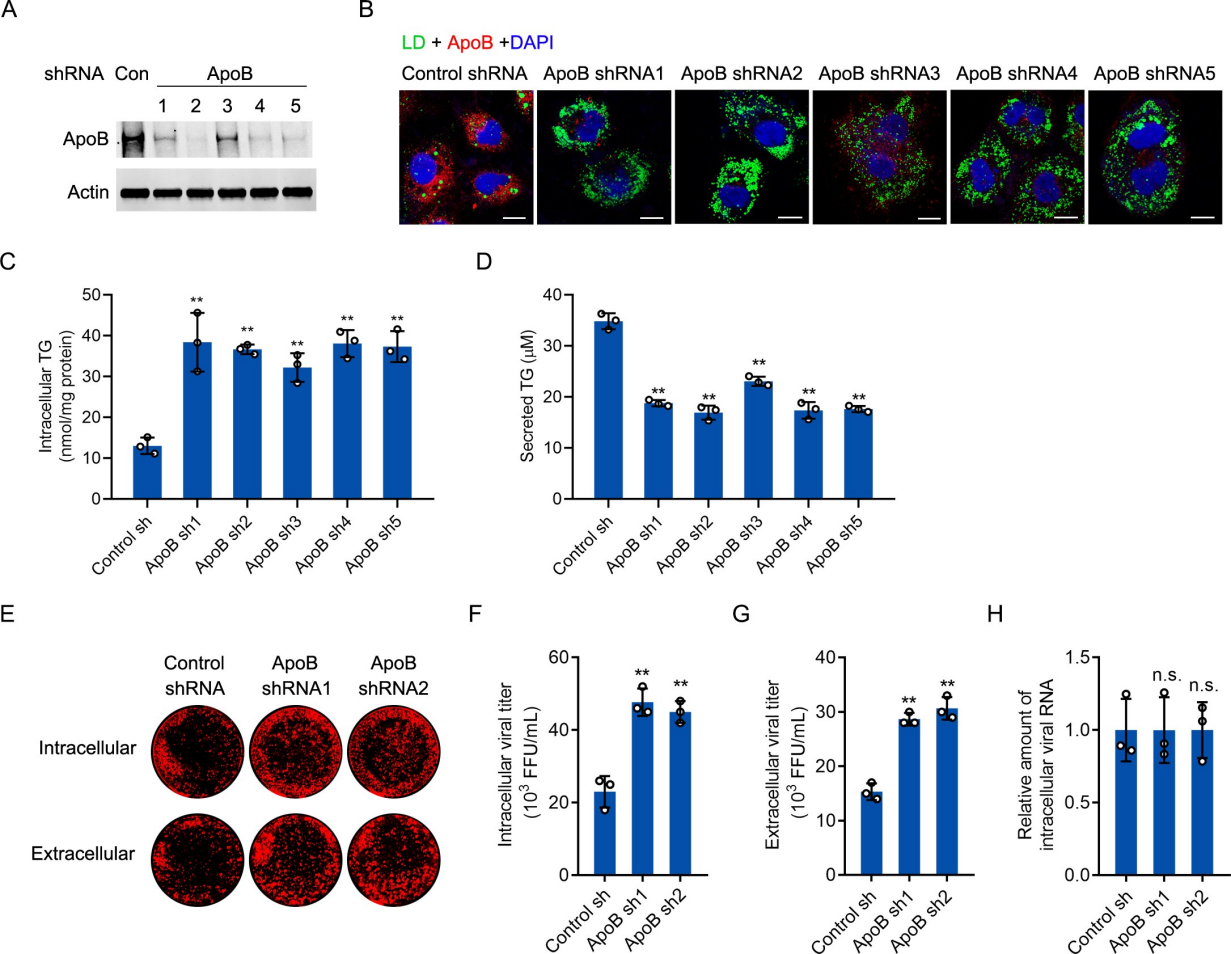
The knockdown of ApoB increased lipid accumulation and promoted HCV production. (A) Huh-7 cells were transduced with ApoB shRNAs. The protein level of ApoB was analyzed by western blotting. Actin was used as the loading control. (B) Huh-7 cells were transduced with ApoB shRNA1-shRNA5. Immunostaining was performed with an anti-ApoB antibody. LDs were stained with BODIPY 493/503. Nuclei were stained with DAPI. Fluorescence signals were visualized by laser confocal microscopy. Scale bars, 10 μM. (C and D) The intracellular and secreted TG concentrations in cells in B were analyzed using a TG quantification kit. TG, triglyceride. (E) Huh-7 cells were transduced with ApoB shRNA1 or shRNA2 for 2 days prior to HCV infection (MOI = 1) for 4 days. Cell lysates and culture supernatants were collected to infect Huh-7 cells. In-cell western blot analysis was performed with an anti-core antibody. Cells were further labeled with IRDye 800-conjugated secondary antibodies and scanned with an Odyssey infrared imaging system. (F and G) Viral titration was performed by immunostaining. (H) The intracellular HCV RNA level was analyzed by real-time PCR. GAPDH was used as the internal control. The statistical significance was determined by unpaired two-tailed Student’s *t*-tests. The data are presented as the means ± SDs of *n* = 3 biological repeats. n.s., not significant. ** *P* < 0.01.

ApoB-containing VLDL particles are reported to participate in HCV assembly [44,45]. Thus, a question is raised: why does HCV induce ApoB degradation if ApoB is involved in viral assembly? We sought to determine the impact of ApoB silencing on HCV infection. We transduced Huh-7 cells with several shRNAs targeting ApoB and found that ApoB knockdown increased the content of cellular LDs and decreased lipid secretion (Fig 8A–8D). ApoB was then knocked down by shRNAs prior to HCV infection, and both the intracellular and extracellular viral titers but not the intracellular viral RNA level increased (Fig 8E–8H). Because an increased lipid content is beneficial for HCV assembly, we hypothesized that knockdown of ApoB increased the cellular lipid content and promoted HCV replication. To further confirm this speculation, we generated ApoB knockout cell lines by CRISPR-Cas9 gene editing. Knockout was validated in five ApoB-deficient cell clones by Sanger sequencing (S1A and S1B Fig). However, the LD content was not increased in ApoB-deficient cells (S1C Fig). After HCV infection, the extracellular viral titer was not changed in these cell lines compared to the parental cells (S1D Fig).

### The overexpression of ApoB-50 decreased LDs and inhibited HCV assembly

**Fig 9:**
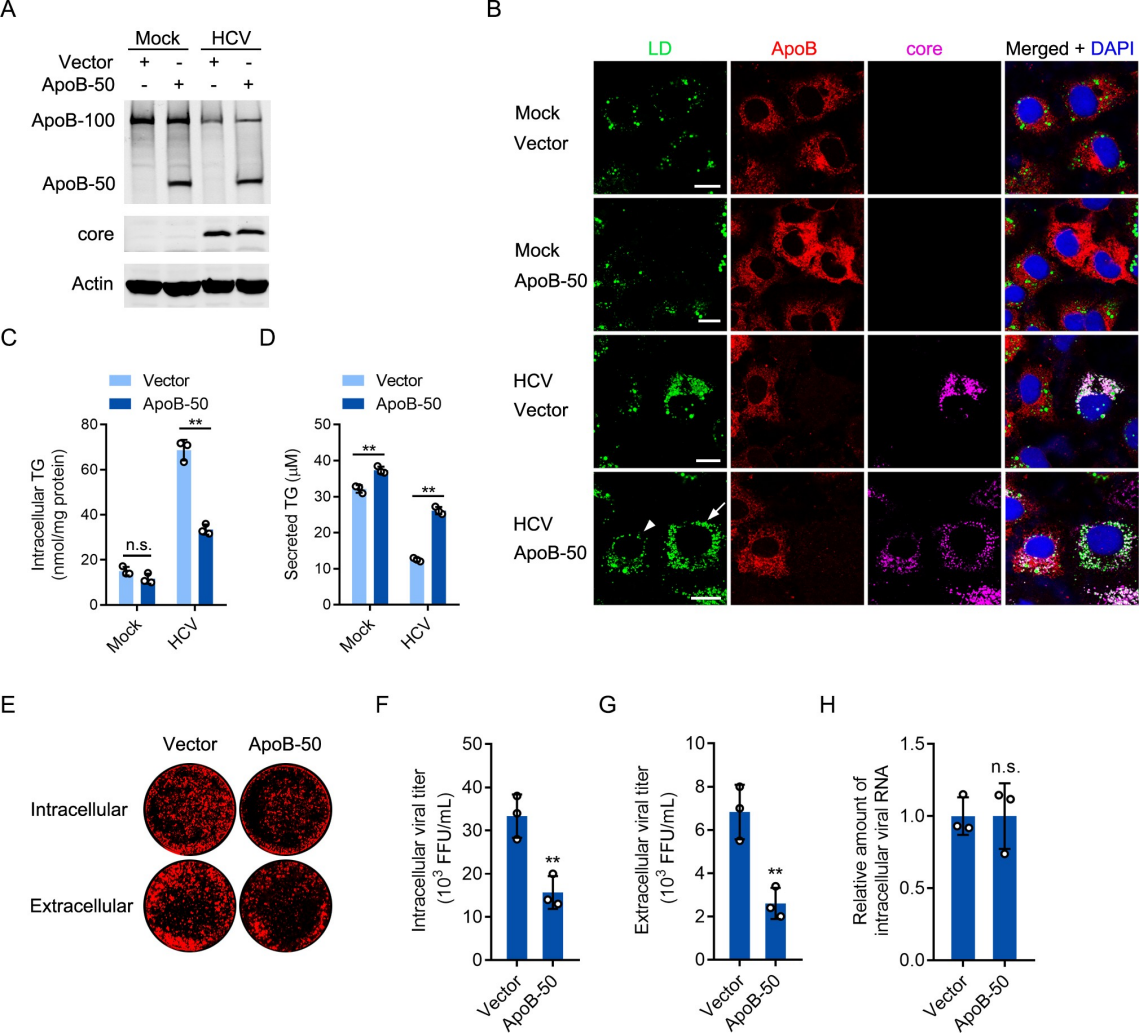
Expression of ApoB-50 decreased lipid accumulation and inhibited HCV production. (A) Huh-7 cells were infected with HCV (MOI = 1) for 2 days prior to transduction with ApoB-50 for 2 days. The protein levels of ApoB and the core were analyzed by western blotting. Actin was used as the loading control. (B) Immunostaining was performed in cells in A with antibodies against ApoB and core. LDs were stained with BODIPY 493/503. Nuclei were stained with DAPI. Fluorescence signals were visualized by laser confocal microscopy. The arrow indicates an HCV-infected cell. The arrowhead indicates an HCV-infected cell transduced with ApoB-50. Scale bars, 10 μM. (C and D) The intracellular and extracellular TG concentrations in cells in A were analyzed using a TG quantification kit. TG, triglyceride. (E-G) Cell lysates and culture supernatants from HCV-infected cells were collected to infect Huh-7 cells. In-cell western blot analysis and viral titration assays were performed. (H) The intracellular HCV RNA level was analyzed by real-time PCR. GAPDH was used as the internal control. The statistical significance was determined by unpaired two-tailed Student’s *t*-tests. The data are shown as the means ± SDs of *n* = 3 biological repeats. n.s., not significant. ** *P* < 0.01.

To further establish the association between the protein level of ApoB and the LD content, we tried to overexpress ApoB in HCV-infected cells. However, ApoB could not be overexpressed because of its large molecular weight. C-terminal truncated ApoB can mediate the assembly and secretion of VLDL [46]. We thus sought to determine whether overexpression of ApoB-50 (amino acids 1–2236 of ApoB) can reduce the LD content in HCV-infected cells (Fig 9A). As shown in Fig 9B to 9D, ApoB-50 overexpression reduced the LD and intracellular triglyceride (TG) contents but increased the level of secreted TG. Moreover, overexpression of ApoB-50 reduced the intracellular and extracellular viral titers but not the intracellular viral RNA level (Fig 9E–9H).

Because inhibition of oxidative stress restored ApoB, we then analyzed whether treatment with antioxidants could influence lipid content and HCV assembly. As shown in S2 Fig, NAC/PDTC treatment slightly decreased the lipid content in mock- and HCV-infected cells. NAC/PDTC slightly increased the level of secreted TG in HCV-infected cells but not in mock-infected cells. HCV RNA and viral titers were significantly decreased by NAC/PDTC treatment.

## Discussion

**Fig 10:**
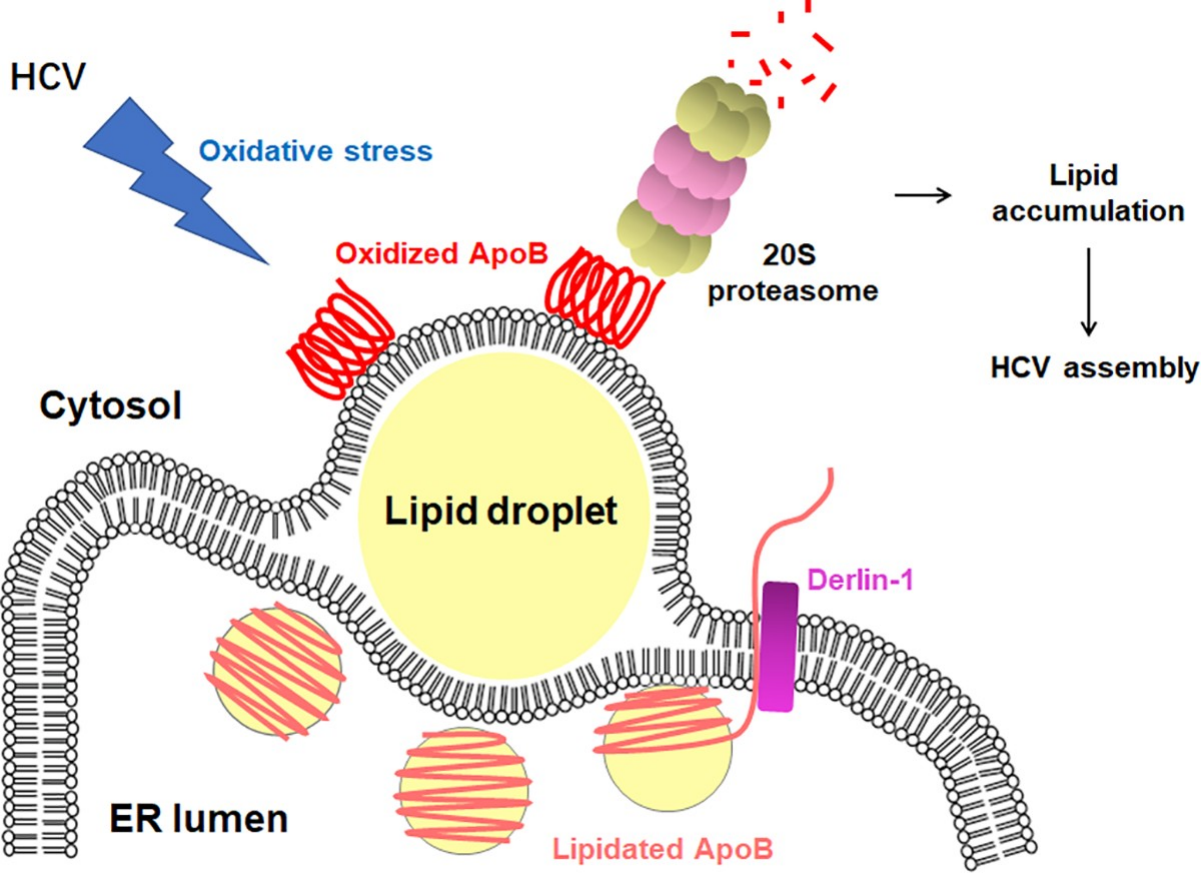
Schematic representation of ApoB degradation in HCV-infected Huh-7 cells. ApoB is retrotranslocated from the ER lumen to cytosolic LDs via a process requiring Derlin-1. HCV infection induces oxidative stress and results in ApoB oxidation. Oxidized ApoB is recognized and degraded by the 20S proteasome. The degradation of ApoB impairs lipid secretion and contributes to lipid accumulation, which might lead to enhanced HCV production.

In this study, we found that ApoB was degraded directly by the 20S proteasome in a ubiquitin-independent manner but not by ERAD or autophagy in HCV-infected Huh-7 cells. HCV-induced oxidative stress induced the oxidation of ApoB, which promoted its recognition and degradation by the 20S proteasome. ApoB was retrotranslocated to ER-associated LDs before degradation via a process requiring Derlin-1. Degradation of ApoB induced by HCV infection contributed to lipid accumulation and benefited viral assembly (Fig 10).

The mechanisms underlying ApoB degradation have been studied in the last two decades. ApoB can be degraded either cotranslationally or after the full-length protein has entered the secretory pathway by ERAD and autophagy[43]. During HCV infection, ApoB degradation can be inhibited by MG-132, but the mechanism of its degradation is incompletely elucidated. Carmine et al. reported that HCV infection upregulated the expression of ferritin heavy chain (FTH1), which inhibited ApoB export from the ER and increased ERAD of ApoB in Huh-7.5.1 cells[21]. In our study, the expression of FTH1 was investigated in mock- and HCV-infected Huh-7 cells (S3 Fig). In contrast with previous results, FTH1 was expressed at a low level in Huh-7 cells, and HCV infection did not increase its expression. Moreover, ApoB was not degraded through the ERAD pathway in our study. Huh-7.5.1 is a Huh-7 cell-derived RIG-I-mutated cell line and is highly permissive for HCV infection [47,48]. Moreover, Huh-7 cells from different sources vary in morphology, cell growth, and permissiveness for HCV infection [49]. The discrepancy between our study and Carmine’s study might be explained by the different cell lines that we used and their different cell sources.

HCV infection can induce complete autophagy through ER stress [22]. We initially investigated whether ApoB is degraded by ERAD or autophagy during HCV infection. However, we found that neither ERAD nor autophagy participated in ApoB degradation. Both ERAD and autophagy require the ubiquitination of substrates. After HCV infection, ApoB was degraded in a ubiquitin-independent manner, indicating that ApoB degradation occurred through other pathways and not through ERAD or autophagy. Interestingly, HCV infection induced a reduction in the level of gp78, which is required for ERAD. Thus, HCV infection apparently suppressed ERAD. The underlying mechanism and biological significance of this effect could be studied in the future.

Because MG-132 inhibits not only proteasome-associated degradation but also the activity of other proteases, the experimental results with MG-132 were not sufficient to conclude that ApoB is degraded by the proteasome in HCV-infected cells. However, experiments with another proteasome inhibitor, bortezomib, and an shRNA targeting the PSMA4 subunit of the 20S proteasome confirmed that ApoB was indeed degraded by the proteasome. Further studies showed that the 20S proteasome degraded ApoB in HCV-infected cells without the need for regulatory particles. Moreover, the degradation of ApoB was mediated not only by the 19S-20S proteasome complex (26S proteasome) but also by the 11S-20S proteasome complex, the PA28γ-20S proteasome complex, and the PA200-20S proteasome complex in mock-infected Huh-7 cells. Interestingly, all of these proteasome regulators contributed to the turnover of ApoB but to different extents.

Oxidative stress induced by H_2_O_2_ has been reported to inactivate the ubiquitin-activating/conjugating system and cause the dissociation of the 20S core from the 19S regulator [33,34]. However, the amount of 20S proteasome was not increased in HCV-infected cells, probably due to the relatively mild oxidative stress induced by HCV compared to that induced by H_2_O_2_. Oxidized proteins can be substrates of the 20S proteasome for degradation. In addition, protein oxidation has been hypothesized to result in partial unfolding and exposure of internal hydrophobic patches that can be recognized by the α subunits of the 20S proteasome. We found that ApoB was oxidized in HCV-infected but not mock-infected cells and that inhibition of ApoB oxidation reversed its degradation. We also found a direct interaction of the PSMA5 and PSMA6 subunits of the 20S proteasome with oxidized ApoB but not with unoxidized ApoB. Notably, the protein level of overexpressed ApoB-50 was not changed in HCV-infected cells. Thus, oxidation of the C-terminal half of ApoB might mediate its recognition and degradation by the 20S proteasome.

Dongsheng Yu et al. found that oxidative stress could inhibit *ApoB* transcription in HepG2 cells [50]. Although HCV infection induced oxidative stress in Huh-7 cells, the mRNA level of *ApoB* was slightly increased at 4 days after infection (Fig 1D). Treatment with antioxidants did not change the mRNA level of *ApoB* in mock- or HCV-infected cells (S4A Fig). We also analyzed the level of *ApoB* mRNA in Huh-7 cells treated with H_2_O_2_. The level of *ApoB* mRNA was increased by H_2_O_2_ at low concentrations but decreased at a higher concentration in Huh-7 cells (S4B Fig).

Mechanisms by which HCV induces lipid accumulation include increased lipogenesis [51,52], impaired lipid secretion [53], and impaired lipolysis and β-oxidation [54,55]. ApoB degradation can result in a decrease in lipid secretion and induce lipid accumulation in noninfected livers [56]. However, whether HCV-induced ApoB degradation contributes to steatosis has not been clarified. Domitrovich et al. found that inhibition of ApoB degradation by ALLN did not inhibit lipid accumulation in HCV subgenomic replicon-harboring cells [57]. They found that HCV induced a decrease in microsomal triglyceride transfer protein (MTP), which is required for lipoprotein assembly and secretion. In our study, we did not determine the expression of MTP, but we found that overexpression of ApoB-50, which contains the essential β1 domain for lipoprotein assembly and secretion, reduced the cellular lipid content and increased the level of secreted lipids. These results suggested that ApoB degradation contributed to steatosis. Inhibition of ApoB degradation by proteasome inhibitors would not repair the damage to ApoB caused by oxidation; in addition, oxidized ApoB might be defective in its lipoprotein assembly function, which might explain why proteasome inhibitors did not decrease lipid accumulation in our study (Fig 6B) and another study [57].

We noted that HCV infection decreased the protein level of ABAC1. Mingxia Liu et la. found that hepatic ABCA1 deficiency was associated with impaired ApoB secretion and enhanced VLDL TG secretion [58]. They hypothesized that altered intracellular trafficking of ApoB resulted in augmented TG addition to nascent VLDL. In our study, the protein levels of ApoB and TG secretion were significantly reduced in HCV-infected cells. The effects resulting from ABCA1 deficiency might be overrode by ApoB deficiency. ABCA1 upregulation could inhibit virus-host cell fusion to inhibit HCV cell entry [59]. ABCA1 deficiency induced by HCV might enhance viral infection. This hypothesis can be studied in the future.

HCV induces oxidative stress, which has been found to affect both virus replication and the progression and severity of HCV infection [60]. ROS has many targets and functions. ROS reportedly suppress HCV replication, and antioxidants enhance HCV RNA replication [61–64]. In contrast, antioxidants could inhibit HCV replication, as previously reported [65,66]. Antioxidants could also inhibit lipid accumulation in hepatocytes by inhibiting lipogenesis [67,68]. Oxidative stress influences several signaling pathways, including PI3K signaling [69]. The PI3K signaling pathways can not only control HCV replication but also modulate lipid metabolism [70]. HCV-induced activation of the transcription factors NF-kB and STAT-3 was associated with ROS [71]. NF-kB and STAT-3 also regulate HCV replication and lipid metabolism[72]. We found that antioxidants slightly decreased cellular lipid contents but significantly inhibited HCV replication (S2 Fig). Given the controversial results reported and the complexity of ROS functions, it was difficult to interpret the results obtained from antioxidant treatment.

Our study showed that knockdown of ApoB induced lipid accumulation and promoted HCV assembly. Overexpression of ApoB-50 reduced lipid contents and impaired HCV assembly. The assembly and release of HCV particles were thought to require ApoB [44,45]. However, other studies revealed that ApoE but not ApoB was required for HCV assembly [73–75]. Jiang and Luo found that ApoB siRNAs had no significant effect on HCV production in Huh-7.5 cells. In our study, knockdown of ApoB induced lipid accumulation. Because an increased lipid content is beneficial for HCV assembly, the changes in HCV assembly might be associated with the changes in lipid contents induced by ApoB expression alteration. Jiang and Luo collected the virus-containing supernatant 24 hours after transfection with ApoB siRNAs but did not analyze the intracellular lipid content. In our study, significant lipid accumulation was observed at 48 hours after transduction with ApoB shRNAs. ApoB siRNA might not induce significant changes in lipids at 24 hours post transfection, which might explain why Jiang and Luo did not observe a significant effect on HCV production.

Fukuhara et al. found that knockout of ApoB in Huh-7 cells did not affect HCV production [74]. We generated ApoB^-/-^ Huh-7 cells by CRISPR-Cas9 gene editing, and we also found that HCV production did not change in ApoB^-/-^ Huh-7 cells. Notably, the LD content was not increased in ApoB-deficient cells, which might explain why HCV production was not affected by ApoB knockout. It has been reported that genetic compensation can be induced by gene deficiency but not gene knockdown [76]. Thus, knockout of ApoB might upregulate other pathways to reverse the accumulation of LDs.

The assembly of ApoB-containing VLDL competed with HCV assembly. ApoB-containing lipoproteins also competed for binding entry factors with HCV on target cells. Overexpression of ApoB-50 might inhibit HCV assembly and viral entry through VLDL assembly and secretion. Therefore, the impact of ApoB knockdown or ApoB-50 overexpression on HCV production might be a pooled effect. However, knockout of ApoB in Huh-7 cells did not affect HCV production, as shown in S2 Fig and as previously reported [74]. The impact of the VLDL assembly pathway on HCV production might have been minimal in our study.

Considering the redundancy of apolipoprotein usage in HCV assembly, we also analyzed the protein levels of other apolipoproteins involved in HCV assembly (ApoA1, ApoC1, and ApoE) [74,75]. ApoE was moderately decreased at day 4 after HCV infection (S5A Fig), which was consistent with a previous report that ApoE is degraded by HCV-induced autophagy [77]. The expression of ApoA1 and ApoC1 is suppressed in hepatic cancer cell lines [74] and can hardly be detected in Huh-7 cells by immunoblotting (S5B Fig). We then analyzed whether overexpression of these apolipoproteins could further enhance HCV assembly while knocking down ApoB. We found that overexpression of ApoA1 or ApoC1 did not change intracellular and extracellular viral titers (S5C and S5D Fig). However, overexpression of ApoE increased intracellular and extracellular viral titers. These results suggested that ApoE was redundant and might play a dominant role in HCV assembly.

In summary, we showed that HCV infection induced the degradation of ApoB by the 20S proteasome through the induction of oxidative stress. Degradation of ApoB contributed to lipid accumulation, which might promote HCV assembly. Our study revealed a novel mechanism for ApoB degradation and lipid accumulation during HCV infection and might suggest new therapeutic targets for hepatic steatosis.

## Materials and methods

### Cell culture

Huh-7 human hepatoma cells were maintained as a stock in our laboratory and cultured in Dulbecco’s modified Eagle’s medium (DMEM) supplemented with 10% fetal bovine serum (FBS) (Thermo Fisher Scientific, USA), 100 units/ml penicillin, and 100 μg/ml streptomycin. The Huh-7.5.1 cell line, a Huh-7 variant highly permissive for HCV replication, was provided by Dr. Francis V. Chisari (The Scripps Research Institute, La Jolla, CA, USA) and grown in DMEM supplemented with 10% FBS and an additional 2 mM L-glutamate (Thermo Fisher Scientific, USA). The HEK293T cell line was purchased from Takara and maintained in DMEM supplemented with 10% FBS.

### Antibodies and reagents

The following antibodies were used in this study: anti-β-actin (A5441, 1:5000 for immunoblotting), anti-FLAG (A8592, 1:5000 for immunoblotting), anti-GST (G7781, 1:2000 for immunoblotting), anti-Derlin-1 (D4443, 1 μg/mL for immunoblotting), anti-calnexin (C4731, 5 μg/mL for immunostaining) (all purchased from Sigma-Aldrich), anti-gp78 (9590S, 1:1000 for immunoblotting), anti-ABCA1 (96292S, 1:1000 for immunoblotting) (both purchased from Cell Signaling Technology), anti-ApoB pAb (ab7616, 5 μg/mL for immunoprecipitation (IP), 1 μg/mL for immunoblotting, and 5 μg/mL for flow cytometric assay), anti-p97 (ab11433, 1 μg/mL for immunoblotting), anti-FTH1 (ab75972, 1 μg/mL for immunoblotting), anti-ADFP (ab108323, 1 μg/mL for immunoblotting), anti-ERp57 (ab13506, 5 μg/mL for immunostaining) (all purchased from Abcam), anti-HCV core protein (sc-57800, 1 μg/mL for immunoblotting, 5 μg/mL for immunostaining and flow cytometric assay), anti-ApoB mAb (sc-13538, 1 μg/mL for immunoblotting), anti-ApoB mAb agarose (sc-13538AC for IP), anti-ApoA1 (sc-376818, 1 μg/mL for immunoblotting), anti-ApoC1 (sc-101263, 1 μg/mL for immunoblotting), and anti-ApoE (sc-13521, 1 μg/mL for immunoblotting) (purchased from Santa Cruz Biotechnology). Corresponding IRDye 680- or 800-labeled secondary antibodies (1:20000 for immunoblotting) were obtained from LI-COR Biosciences. The fluorescence-labeled secondary antibodies used for immunostaining were purchased from Jackson ImmunoResearch. MG-132 (S2619), bortezomib (S1013), PD151746 (S7424), and E64d (S7393) were purchased from Selleck Chemicals. DBeQ (SML0031), wortmannin (12–338), and Eer I (E1286) were purchased from Sigma-Aldrich. BODIPY 493/503 (D3922, 0.5 μg/mL for staining), 4′,6-diamidino-2-phenylindole (DAPI) (D1306, 300 ng/mL for staining), and Lipofectamine RNAiMAX Transfection Reagent (13778075) were purchased from Thermo Fisher Scientific. Purified human 20S proteasome (E-360) was purchased from R&D Systems.

### siRNA transfection

siRNAs targeting p97 (sc-37187) were purchased from Santa Cruz Biotechnology. siRNAs targeting Derlin-1 were purchased from RiboBio (Guangzhou, China). These siRNAs were transfected into cells at a final concentration of 50 nM with Lipofectamine RNAiMAX Transfection Reagent according to the manufacturer’s instructions. The efficiency of gene knockdown was determined by immunoblotting.

### HCV JFH1 strain (genotype 2a) production, titration, and infection

The plasmid pcDNA6/TR-Tight/JFH1-FL/AR harboring JFH1 strain cDNA was kindly provided by Dr. Guangxiang Luo (Department of Microbiology, School of Medicine, University of Alabama-Birmingham) [78]. The methods used for HCV production, titration, and infection were described previously [22].

### Lentiviral packaging, titration, and transduction

For gene knockdown experiments, plasmids containing ApoB (TRCN0000003738 to TRCN0000003742), gp78 (TRCN0000003375), PSMA4 (TRCN0000003884), PSMD4 (TRCN0000273213), PSME2 (TRCN0000365036), PSME3 (TRCN0000290025), PSME4 (TRCN0000158223), and a nontargeting negative control (SHC002) shRNA were obtained from the MISSION TRC library (Sigma-Aldrich). For overexpression, the p97 and ApoB-50 sequences were cloned into the pLVX vector (Takara, Japan). For lentiviral packaging, 6 μg of lentiviral packaging mix (SHP001, Sigma-Aldrich) and 4 μg of the plasmids mentioned above were cotransfected into 6×10^6^ HEK293T cells with 30 ng of polyethyleneimine (PEI​) (408727, Sigma-Aldrich). The lentivirus-containing supernatants were collected, filtered, and stored at -80°C as aliquots. The methods used for lentiviral titration and transduction were described previously [79].

### SDS-PAGE and immunoblotting

Cells were lysed with radioimmunoprecipitation assay (RIPA) buffer (50 mM Tris-HCl, pH 7.5; 150 mM NaCl; 5 mM EDTA; 1% Nonidet-P40; 0.5% sodium deoxycholate; and 0.1% SDS) supplemented with protease and phosphatase inhibitor cocktails (Roche). For analysis of gp78 and Derlin-1 expression, cells were lysed with 1× loading buffer (50 mM Tris-HCl, pH 7.5; 1% SDS; 1% glycerol; and 1% 2-mercaptoethanol) supplemented with benzonase (E1014, Sigma-Aldrich). Equal amounts of total protein were electrophoresed on an SDS-PAGE gel and then transferred to a nitrocellulose membrane (Pall, USA). For analysis of ApoB expression, cell lysates were supplemented with 4× lithium dodecyl sulfate (LDS) loading buffer and electrophoresed on a NuPAGE 3–8% Tris-acetate gel (EA03785, Thermo Fisher Scientific). After being blocked with 5% nonfat milk in phosphate-buffered saline (PBS; pH 7.4), membranes were incubated first with primary antibodies and then with the IRDye-labeled secondary antibodies listed above. Finally, membranes were scanned with an Odyssey infrared imaging system (Li-COR Biotechnology).

### Isolation of LDs and microsomes

Isolation of LDs and microsomes was performed using an Endoplasmic Reticulum Isolation Kit (ER0100, Sigma-Aldrich) with some modification. Briefly, cells were resuspended in 1× Hypotonic Extraction Buffer supplied by the kit and homogenized using a Dounce homogenizer. The samples were centrifuged at 12,000 g for 15 minutes at 4°C. The thin floating lipid layer was the LD fraction. The LD fractions were washed twice with 1× Isotonic Extraction Buffer and supplemented with 4× LDS loading buffer. The supernatants without lipid layers were transferred and centrifuged for 60 minutes at 100,000 g in an ultracentrifuge at 4°C. The resulting pellets were the microsomal fractions and were dissolved in 1× LDS loading buffer. After heating for 10 minutes at 70°C, the LD and microsomal fractions were analyzed by immunoblotting.

### Purification of GST fusion proteins and GST pull-down assay

GST fusion proteins were expressed in *E*. *coli* BL21 and induced with 0.1 mM isopropyl-1-thio-β-D-galactopyranoside (IPTG) for 12 hours at 16°C. The cells were lysed by sonication, and the lysates were incubated with glutathione-Sepharose 4B beads (GE Biosciences) by rocking at 4°C for 1 hour. Huh-7 cells were lysed with IP buffer (25 mM Tris-HCl pH 7.4, 150 mM NaCl, 1% NP-40, 1 mM EDTA, 5% glycerol). Whole-cell lysates were added to the beads and incubated by rocking at 4°C overnight. The beads were washed three times with IP buffer, dissolved in 2× LDS loading buffer and heated for 10 minutes at 70°C. After centrifugation, the supernatant was analyzed by immunoblotting.

### IP under nondenaturing condition

For IP under nondenaturing conditions, cells were harvested and then lysed in IP buffer supplemented with a complete protease inhibitor cocktail (Roche). Whole-cell lysates were incubated with mouse anti-ApoB mAb agarose beads at 4°C overnight. Immunoprecipitates were extensively washed with IP buffer and subjected to subsequent analysis.

### IP under denaturing condition

For IP under denaturing conditions, cells were harvested and then lysed in RIPA buffer supplemented with 10% LDS and protease and phosphatase inhibitor cocktails. The lysates were heated for 10 minutes at 70°C and then diluted 4 times with IP buffer. Whole-cell lysates were incubated with mouse anti-ApoB mAb agarose beads at 4°C overnight. Immunoprecipitates were extensively washed with IP buffer and subjected to subsequent analysis.

### Protein oxidation analysis

ApoB was immunoprecipitated under denaturing conditions without LDS. The immunoprecipitates were subjected to analysis of ApoB oxidation. ApoB oxidation was analyzed using an OxyBlot Protein Oxidation Detection Kit (S7150, Sigma-Aldrich) according to the manufacturer’s instructions. The carbonyl groups in the protein side chains are derivatized to 2,4-dinitrophenylhydrazone (DNP) by reaction with 2,4-dinitrophenylhydrazine (DNPH). The reaction mixtures were dissolved in 2× LDS loading buffer and heated for 10 minutes at 70°C. The samples were subjected to immunoblotting.

### 20S proteasome degradation assay

ApoB was immunoprecipitated under nondenaturing conditions. Equal amounts of immunoprecipitates were incubated with purified human 20S proteasome at 37°C in HEPES buffer (50 mM HEPES, pH 7.5, 1 mM DTT and 0.02% SDS) for the indicated timepoints. The reaction mixtures were dissolved in 2× LDS loading buffer and heated for 10 minutes at 70°C. After centrifugation, the supernatant was analyzed by immunoblotting.

### In-gel peptidase activity assay for 26S and 20S proteasomes

Cell lysates were separated on a native-PAGE gel. For the analysis of the proteolytic activity of the 26S proteasome, the gel was incubated with 100 μM suc-LLAV-AMC in developing buffer (50 mM Tris, pH 7.5, 150 mM NaCl, 5 mM MgCl_2_, and 1 mM ATP) for 30 minutes. For the analysis of the proteolytic activity of the 20S proteasome, the gel was stained with 100 μM suc-LLAV-AMC in developing buffer in the presence of 0.02% SDS for 30 minutes. The stained gel was analyzed using a UV trans-illuminator at 365 nm wavelength.

### In-cell western assay

An in-cell western assay was used for viral titration. Cells cultured on 96-well plates were infected with HCV. After 72 hours, cells were fixed for 15 minutes with 4% paraformaldehyde and permeabilized for 20 minutes at room temperature with 0.25% Triton X-100 in PBS. After incubation with 5% BSA in PBS for 2 hours, cells were incubated with an anti-core antibody overnight at 4°C. Cells were further stained with IRDye 800-conjugated secondary antibodies and scanned with an Odyssey infrared imaging system.

### Real-time PCR

Total RNA from cell samples was isolated by using TRIzol reagent (Thermo Fisher Scientific) and treated with RNase-free DNase I (New England Biolabs). Total RNA was reverse transcribed using a High-Capacity cDNA Reverse Transcription Kit (4368814, Thermo Fisher Scientific). The resulting cDNA was used for real-time quantitative PCR with Power SYBR Green PCR Master Mix (Thermo Fisher Scientific). GAPDH was used as the internal control. The sequences of the primers were as follows:

GAPDH-F: 5´-TGGGCTACACTGAGCACCAG-3´

GAPDH-R: 5´-AAGTGGTCGTTGAGGGCAAT-3´

HCV-F: 5´-TCTGCGGAACCGGTGAGTA-3´

HCV-R: 5´-TCAGGCAGTACCACAAGGC-3´

ApoB-F: 5´-TGCTCCACTCACTTTACCGTC-3´

ApoB-R: 5´-TAGCGTCCAGTGTGTACTGAC-3´

### TG level quantification

The TG concentration was measured using a TG quantification kit (MAK266) from Sigma-Aldrich. For intracellular TG quantification, cultured cells (1×10^6^) were homogenized in 100 μl of 5% Nonidet-P40 Substitute solution. Samples were slowly heated to 100°C to solubilize all TGs and diluted before measurement. The cell culture supernatants were measured directly. The TG concentration was measured and calculated according to the manufacturer’s manual.

### Immunofluorescence and confocal microscopy

Cells cultured on coverslips were fixed for 15 minutes with 4% paraformaldehyde and permeabilized for 20 minutes at room temperature with 0.25% Triton X-100 in PBS. After incubation with 5% BSA in PBS for 2 hours, the cells were incubated with primary antibodies overnight at 4°C and were then labeled with fluorescence-labeled secondary antibodies. LDs were stained with BODIPY 493/503. Nuclei were stained with DAPI. Images were acquired and analyzed using a TCS SP5 laser scanning confocal microscope and companion software (Leica Microsystems).

### Flow cytometry

Huh-7 cells were harvested and fixed for 10 minutes with 4% paraformaldehyde followed by permeabilization for 10 minutes at room temperature with 0.1% Triton X-100 in PBS supplemented with 5% FBS. Subsequently, the cells were incubated with mouse anti-core (5 μg/mL) and goat anti-ApoB (5 μg/mL) antibodies diluted with staining buffer (PBS, 0.1% saponin, 5% BSA) overnight at 4°C and were then labeled with FITC-conjugated anti-goat IgG (5 μg/mL) and PE-Cy7-conjugated anti-mouse IgG_1_ (5 μg/mL) antibodies for 1 hour at room temperature. LDs were stained with HCS LipidTOX Deep Red Neutral Lipid Stain (H34477, 0.5 μg/mL for staining, Thermo Fisher Scientific). Cells were analyzed using a BD FACS Canto II Cell Analyzer (BD Biosciences). The data were analyzed using FlowJo 10.0 software (BD Biosciences).

### CRISPR/Cas9-mediated genome editing

ApoB gene-knockout Huh-7 cell lines were generated by using CRISPR/Cas9 gene-editing technology. Guide RNA sequences were cloned into the lentiCRISPRv2 vector (obtained from Addgene). Huh-7 cells were transfected with the lentiCRISPRv2 vector and selected with puromycin (1 μg/mL) for 7 days. Cell clones were isolated, and the genomic DNA surrounding the edited sites was analyzed by Sanger sequencing.

### Statistical analysis

All data are presented as the means ± SDs. The statistical analyses were performed using GraphPad Prism 8.0 software. The statistical significance was determined using an unpaired two-tailed Student’s *t*-test for comparisons between two groups. The level of statistical significance is indicated in the figure legends as *P < 0.05 and **P < 0.01.

The numerical data used in all figures are included in S1 Data.

## Supporting information

S1 Fig ApoB knockout did not affect cellular lipid and HCV production. (A) Huh-7 cells were transduced with guide RNAs and Cas9 and treated with puromycin for 7 days. Cell clones were isolated, and the level of ApoB was analyzed by western blotting. (B) The ApoB gene sequences of randomly selected clones were analyzed by Sanger sequencing. (C) Immunostaining was performed with an anti-ApoB antibody on ApoB^-/-^ cell clones. LDs were stained with BODIPY 493/503. Nuclei were stained with DAPI. Fluorescence signals were visualized by laser confocal microscopy. Scale bars, 10 μM. (D) ApoB^-/-^ cell clones were infected with HCV. Extracellular viral titers were analyzed. The data are shown as the means ± SDs of *n* = 3 biological repeats. The statistical significance was determined by unpaired two-sided Student’s *t*-tests. n.s., not significant.
(TIF)

S2 Fig Impacts of antioxidants on lipid contents and HCV assembly. (A) Huh-7 cells were transduced with guide RNAs and Cas9 and treated with puromycin for 7 days. Cell clones were isolated, and the level of ApoB was analyzed by western blotting. (B) The ApoB gene sequences of randomly selected clones were analyzed by Sanger sequencing. (C) Immunostaining was performed with an anti-ApoB antibody on ApoB^-/-^ cell clones. LDs were stained with BODIPY 493/503. Nuclei were stained with DAPI. Fluorescence signals were visualized by laser confocal microscopy. Scale bars, 10 μM. (D) ApoB^-/-^ cell clones were infected with HCV. Extracellular viral titers were analyzed. The data are shown as the means ± SDs of *n* = 3 biological repeats. The statistical significance was determined by unpaired two-sided Student’s *t*-tests. n.s., not significant.
(TIF)

S3 Fig Expression of FTH1 in Huh-7 cells after HCV infection. Huh-7 cells were infected with HCV (MOI = 1). The protein level of FTH1 was analyzed by western blotting. HeLa cells were transfected with GFP-, FTH1-, or Flag-tagged FTH1 (as the positive controls for FTH1). Actin was used as the loading control. * Nonspecific bands
(TIF)

S4 Fig Impact of antioxidants or H_2_O_2_ on the mRNA level of *ApoB*. (A) Huh-7 cells were infected with HCV (MOI = 1) for 4 days followed by NAC (1 mM) and PDTC (100 μM) treatment for 24 hours. The mRNA level of ApoB was analyzed by qPCR. (B) Huh-7 cells were treated with H2O2 for 24 hours. The mRNA level of ApoB was analyzed by qPCR. The results are presented as fold changes in the mRNA level of ApoB relative to that of GAPDH. The data are shown as the means ± SDs of n = 3 biological repeats. The statistical significance was determined by unpaired two-tailed Student’s t-tests. n.s., not significant. * P < 0.05. ** P < 0.01.
(TIF)

S5 Fig Impacts of other apolipoproteins on HCV assembly. (A) Huh-7 cells were infected with HCV (MOI = 1). The protein levels of ApoE and HCV core proteins were analyzed by western blotting at the indicated timepoints after infection. Actin was used as the loading control. (B) Huh-7 cells were transduced with ApoB shRNA1 for 1 day prior to HCV infection (MOI = 1). The cells were transduced with Myc/Flag-tagged ApoA1, ApoC1, and ApoE at 2 days post HCV infection. The levels of ApoA1, ApoC1, and ApoE were analyzed at 4 days post infection. Actin was used as the loading control. (C and D) Intracellular and extracellular viral titers in cells in B were analyzed. The data are shown as the means ± SDs. The statistical significance was determined by unpaired two-tailed Student’s *t*-tests. n.s., not significant. * *P* < 0.05. ** *P* < 0.01.
(TIF)

S1 Data The numerical data used in all figures. (XLSX)
