# Supplementary figures and images for: Hepatitis C virus induces oxidation and degradation of apolipoprotein B to enhance lipid accumulation and promote viral production
Source: PLoS Pathog. 2021 Sep 7;17(9):e1009889. doi: 10.1371/journal.ppat.1009889 (PMC8448335; doi:10.1371/journal.ppat.1009889)

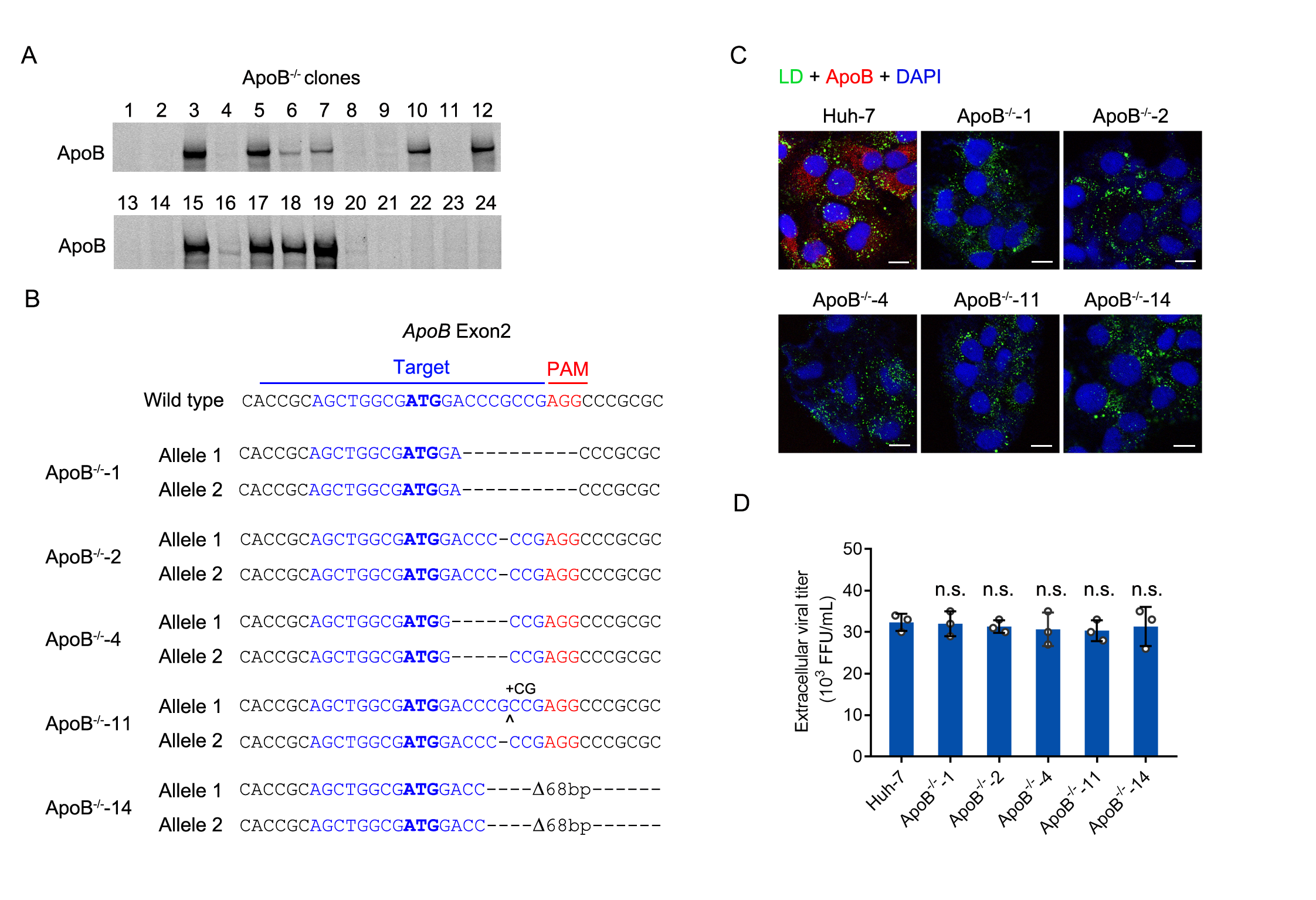

Supplement: S1 Fig — (A) Huh-7 cells were transduced with guide RNAs and Cas9 and treated with puromycin for 7 days. Cell clones were isolated, and the level of ApoB was analyzed by western blotting. (B) The ApoB gene sequences of randomly selected clones were analyzed by Sanger sequencing. (C) Immunostaining was performed with an anti-ApoB antibody on ApoB-/- cell clones. LDs were stained with BODIPY 493/503. Nuclei were stained with DAPI. Fluorescence signals were visualized by laser confocal microscopy. Scale bars, 10 μM. (D) ApoB-/- cell clones were infected with HCV. Extracellular viral titers were analyzed. The data are shown as the means ± SDs of n = 3 biological repeats. The statistical significance was determined by unpaired two-sided Student’s t-tests. n.s., not significant. (TIF) [file ppat.1009889.s001.tif]

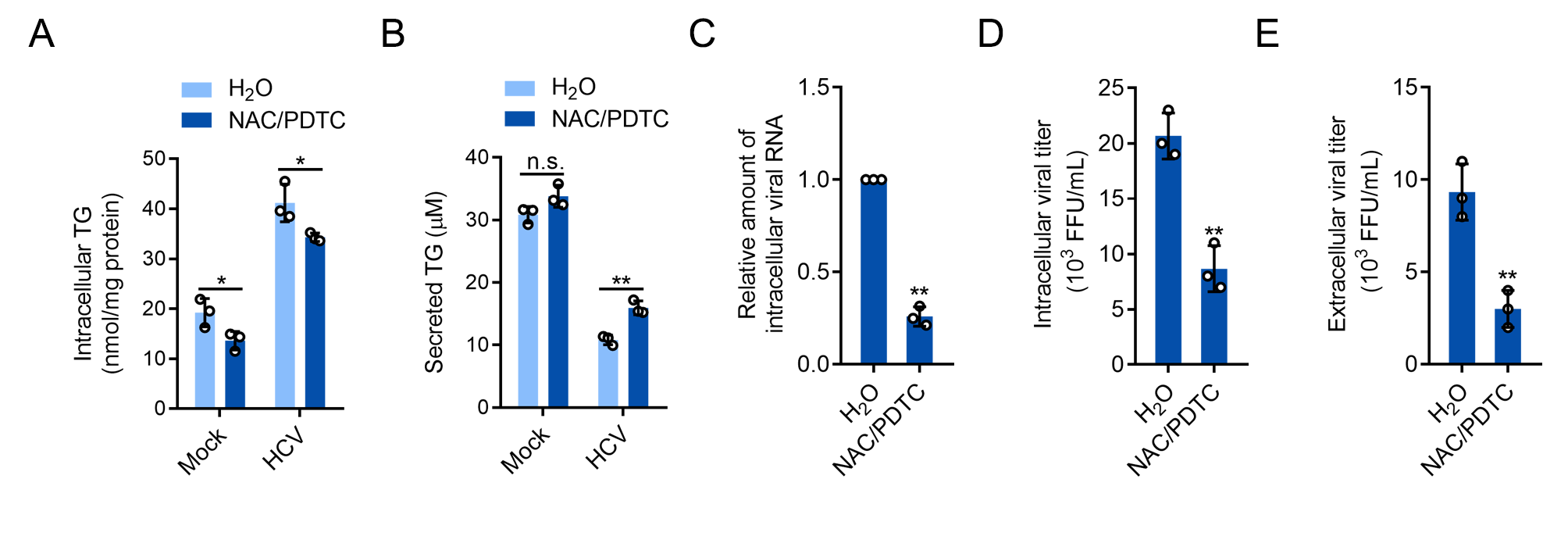

Supplement: S2 Fig — (A) Huh-7 cells were transduced with guide RNAs and Cas9 and treated with puromycin for 7 days. Cell clones were isolated, and the level of ApoB was analyzed by western blotting. (B) The ApoB gene sequences of randomly selected clones were analyzed by Sanger sequencing. (C) Immunostaining was performed with an anti-ApoB antibody on ApoB-/- cell clones. LDs were stained with BODIPY 493/503. Nuclei were stained with DAPI. Fluorescence signals were visualized by laser confocal microscopy. Scale bars, 10 μM. (D) ApoB-/- cell clones were infected with HCV. Extracellular viral titers were analyzed. The data are shown as the means ± SDs of n = 3 biological repeats. The statistical significance was determined by unpaired two-sided Student’s t-tests. n.s., not significant. (TIF) [file ppat.1009889.s002.tif]

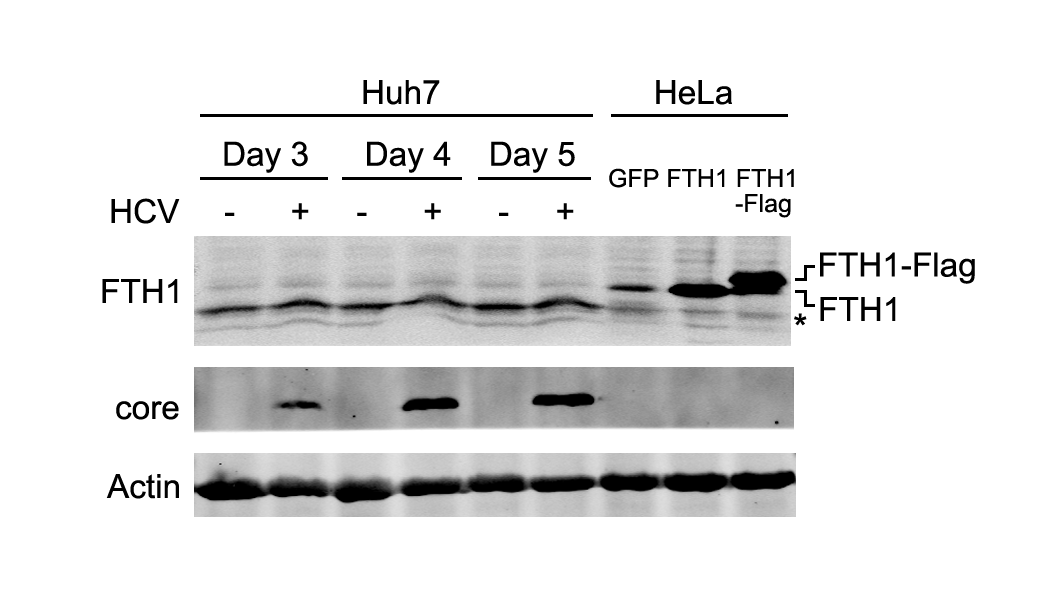

Supplement: S3 Fig — Huh-7 cells were infected with HCV (MOI = 1). The protein level of FTH1 was analyzed by western blotting. HeLa cells were transfected with GFP-, FTH1-, or Flag-tagged FTH1 (as the positive controls for FTH1). Actin was used as the loading control. * Nonspecific bands (TIF) [file ppat.1009889.s003.tif]

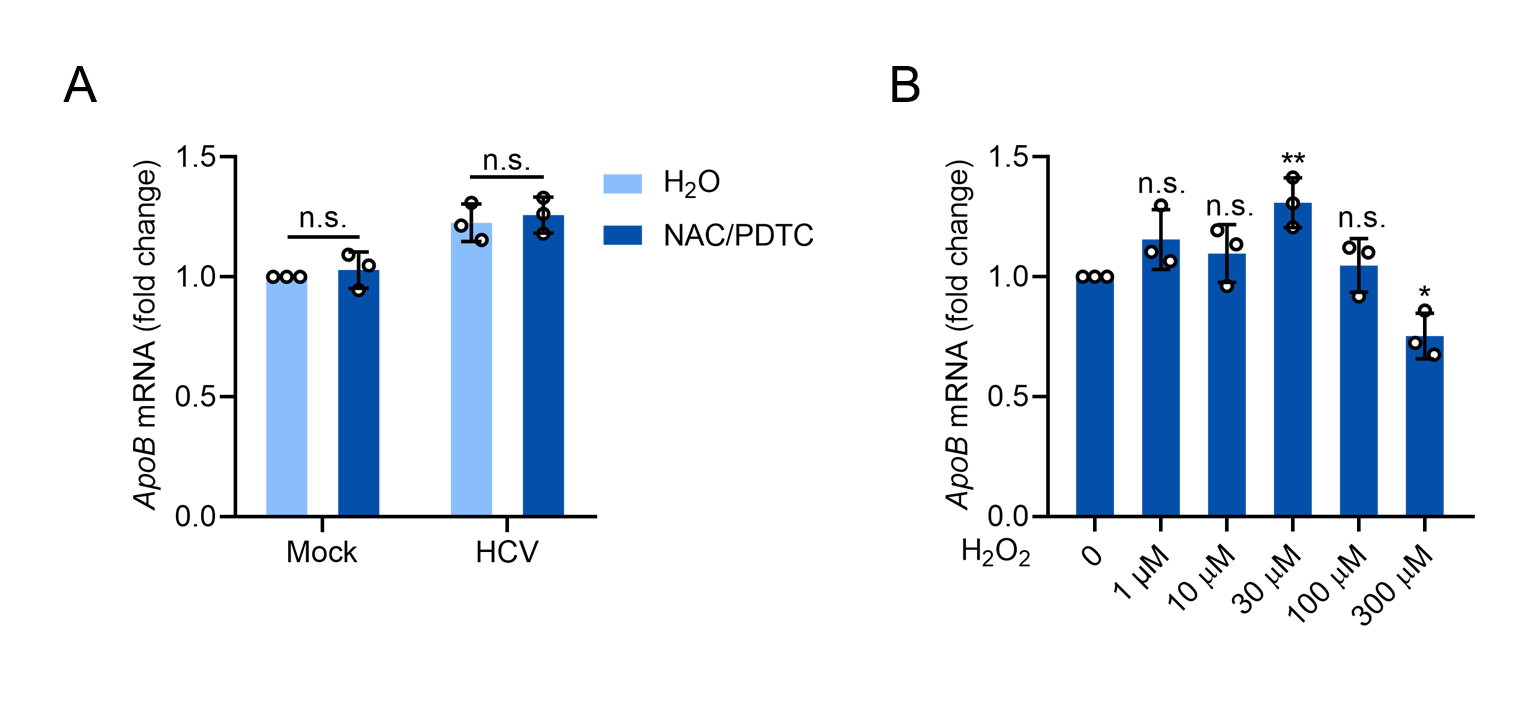

Supplement: S4 Fig — (A) Huh-7 cells were infected with HCV (MOI = 1) for 4 days followed by NAC (1 mM) and PDTC (100 μM) treatment for 24 hours. The mRNA level of ApoB was analyzed by qPCR. (B) Huh-7 cells were treated with H2O2 for 24 hours. The mRNA level of ApoB was analyzed by qPCR. The results are presented as fold changes in the mRNA level of ApoB relative to that of GAPDH. The data are shown as the means ± SDs of n = 3 biological repeats. The statistical significance was determined by unpaired two-tailed Student’s t-tests. n.s., not significant. * P < 0.05. ** P < 0.01. (TIF) [file ppat.1009889.s004.tif]

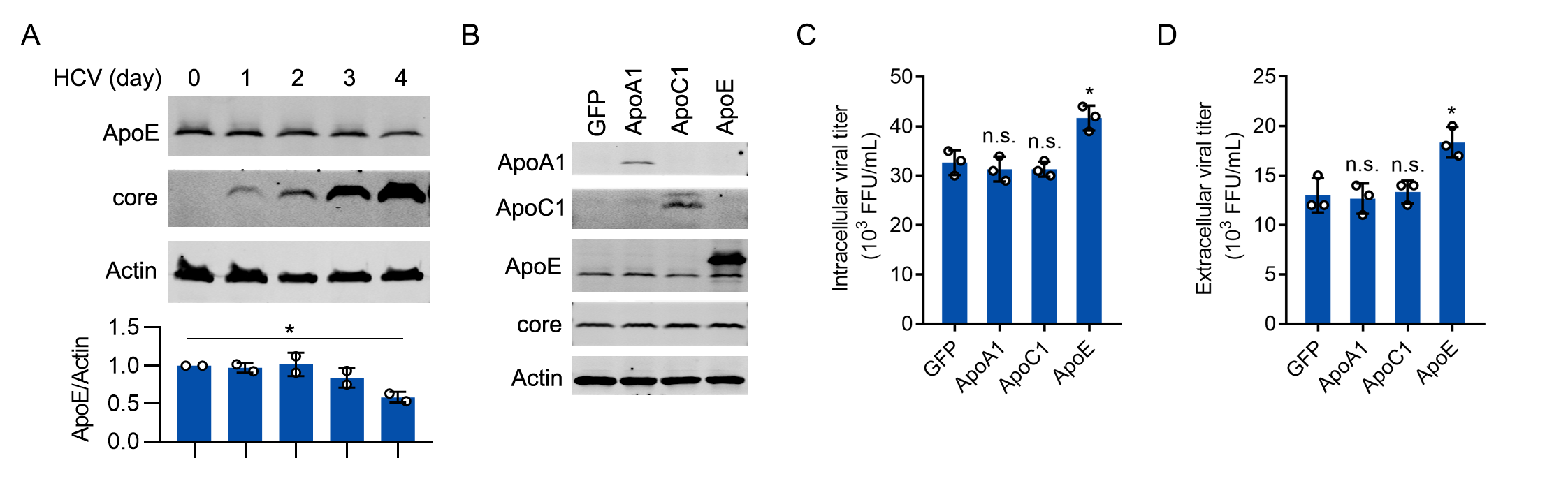

Supplement: S5 Fig — (A) Huh-7 cells were infected with HCV (MOI = 1). The protein levels of ApoE and HCV core proteins were analyzed by western blotting at the indicated timepoints after infection. Actin was used as the loading control. (B) Huh-7 cells were transduced with ApoB shRNA1 for 1 day prior to HCV infection (MOI = 1). The cells were transduced with Myc/Flag-tagged ApoA1, ApoC1, and ApoE at 2 days post HCV infection. The levels of ApoA1, ApoC1, and ApoE were analyzed at 4 days post infection. Actin was used as the loading control. (C and D) Intracellular and extracellular viral titers in cells in B were analyzed. The data are shown as the means ± SDs. The statistical significance was determined by unpaired two-tailed Student’s t-tests. n.s., not significant. * P < 0.05. ** P < 0.01. (TIF) [file ppat.1009889.s005.tif]
